# Complete Description of the LaCl_3_–NaCl Melt Structure and the Concept of a Spacer Salt That Causes Structural Heterogeneity

**DOI:** 10.1021/jacs.2c09987

**Published:** 2022-11-15

**Authors:** Matthew S. Emerson, Shobha Sharma, Santanu Roy, Vyacheslav S. Bryantsev, Alexander S. Ivanov, Ruchi Gakhar, Michael E. Woods, Leighanne C. Gallington, Sheng Dai, Dmitry S. Maltsev, Claudio J. Margulis

**Affiliations:** † Department of Chemistry, The University of Iowa, Iowa City, Iowa 52242, United States; ‡ Chemical Sciences Division, Oak Ridge National Laboratory, Oak Ridge, Tennessee 37831, United States; § Pyrochemistry and Molten Salt Systems Department, Idaho National Laboratory, Idaho Falls, Idaho 83415, United States; ∥ X-ray Science Division, Advanced Photon Source, Argonne National Laboratory, Argonne, Lemont, Illinois 60439, United States; ⊥ Department of Chemistry, University of Tennessee, Knoxville, Tennessee 37996, United States

## Abstract

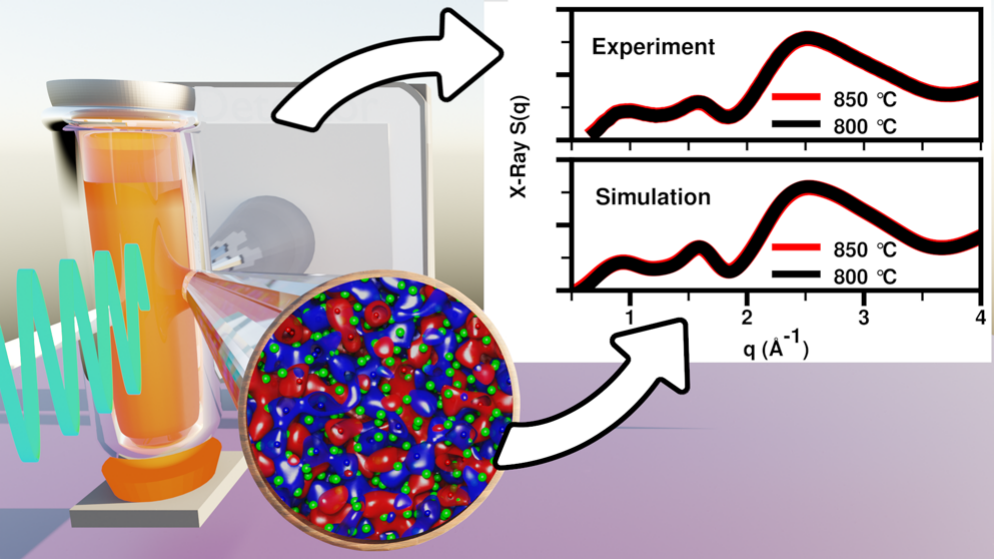

Lanthanides are important fission products in molten salt reactors, and understanding their structure and that of their mixtures is relevant to many scientific and technological problems including the recovery and separation of rare earth elements using molten salt electrolysis. The literature on molten salts and specifically on LaCl_3_ and LaCl_3_–NaCl mixtures is often fragmented, with different experiments and simulations coinciding in their explanation for certain structural results but contradicting or questioning for others. Given the very practical importance that actinide and lanthanide salts have for energy applications, it is imperative to arrive at a clear unified picture of their local and intermediate-range structure in the neat molten state and when mixed with other salts. This article aims to unequivocally answer a set of specific questions: is it correct to think of long-lived octahedral coordination structures for La^3+^? What is the nature as a function of temperature of networks and intermediate-range order particularly upon dilution of the trivalent ion salt? Is the so-called scattering first sharp diffraction peak (FSDP) for neat LaCl_3_ truly indicative of intermediate-range order? If so, why is there a new lower-*q* peak when mixed with NaCl? Are X-ray scattering and Raman spectroscopy results fully consistent and easily described by simulation results? We will show that answers to these questions require that we abandon the idea of a most prominent coordination state for M^3+^ ions and instead think of multiple competing coordination states in exchange due to significant thermal energy in the molten state.

## Introduction

Although the literature on molten salts is significant and spans over a century, the prospect of using them for cleaner and safer energy harvesting and processing to generate electricity has resulted in a massive spike in interest recently.^1−3^ The coordination of ions influences reactivity, including for corrosion and the complex cascade of radiation-driven processes occurring in nuclear reactors.^3−6^ Consequently, a highly desirable objective is to gain complete and consistent understanding of (1) the speciation of ions, (2) the heterogeneity of speciation, and (3) intermediate-range structural correlations that go beyond the arrangement of nearest neighbors in complex multi-ion melts. An added bonus is that speciation information, and its heterogeneity will serve as precious input to simpler thermodynamic models that compute complex salt phase diagrams.^7−12^

Understanding the properties of lanthanides in chloride melts is highly desirable for a variety of cases including fission products and the recovery of rare earth metals by molten salt electrolysis.^13−17^ In addition, LaCl_3_ has long been studied as a structural analogue to UCl_3_^4,18,19^ and more experimental data exists for LaCl_3_ than UCl_3_, partly due to its lower cost and ease of handling. Mixtures of LaCl_3_ in alkali halides also serve as excellent surrogate systems for understanding UCl_3_ fuel salt mixtures for molten salt nuclear reactors (MSRs).^3,20^ Iwadate^21^ published an extensive review on the structure of molten salts and thoroughly discussed the state of the art at the time on MCl_3_-containing salts. A highlight of Iwadate’s article^21^ is the possibility of the existence of dimeric or polymeric octahedral ion complexes that are corner- or edge-shared associated with MCl_6_^3–^, M_2_Cl_10_^4–^, or larger aggregates. Early X-ray diffraction studies of NdCl_3_,^22^ CeCl_3_,^23^ PrCl_3_,^24^ DyCl_3_,^25^ and ErCl_3_^26^ also came to the conclusion that MCl_3_ salts all contained octahedral MCl_6_^3–^ complexes. Yet, these findings were not without some controversy, as at the time, modern polarizable ion models (PIMs) parameterized to include MCl_3_ salts^27−29^ such as ScCl_3_, YCl_3_, TbCl_3_, and LaCl_3_ in cases resulted in integrated pair distribution functions (PDFs) in which the coordination numbers were simply too large for the prevalent picture of octahedral complexes or aggregates apparently supported by Raman experiments.^30^ X-ray absorption fine structure (XAFS)^4,31−33^ and X-ray^24,34^ scattering data on the coordination structure of La^3+^ were also reported as difficult to reconcile,^21^ given that XAFS^4,31^ suggested larger than the originally postulated octahedral coordination. The interpretation by Madden and co-workers^4,19,27−29,35,36^ of the coordination of La^3+^ including their calculation of Raman spectra is, in our view, significantly more nuanced and accurate, as it highlights that the six-coordinate structure should only be expected at a low La^3+^ concentration. Their work suggests that the coordination of La^3+^ is concentration-dependent and that octahedral coordination in the melt is not really required to reproduce the experimental Raman features well; in fact, given how broad the spectral features are, these do not appear to change significantly with the change in coordination that is concomitant with a change in concentration.^4,29,35^ We will see that this correct view of the problem needs to be expanded to consider multiple coordination states within a given melt that have similar free energies with barriers that are easily accessible at the temperatures where these systems are in the molten state.

There is also the interesting issue of a reported first sharp diffraction peak (FSDP) at about 1 Å^–1^ for neat LaCl_3_ in neutron scattering experiments.^37,38^ This peak has been generally ascribed to intermediate-range order,^34,39−41^ and for the smaller and more polarizing M^3+^ ions, this interpretation is likely accurate.^42,43^ For LaCl_3_, the peak is simply caused by incomplete cancellation of the peaks and antipeaks associated with charge alternation and is not really indicative of order beyond that, which characterizes all salts and ionic liquids. The spectral feature is not present in X-ray due to the different contrast compared to neutrons. The work we present here including for mixtures of LaCl_3_ and NaCl as well as the temperature dependence of the so-called prepeak or FSDP suggests that, at least for the larger M^3+^ ions such as La^3+^ and U^3+^, this idea of a small peak at a low *q* value always being associated with intermediate-range order should be revisited and refined. Interestingly, we will show that a bonafide prepeak associated with intermediate-range order does occur for these systems but only in the presence of a lower formal charge “spacer salt”. When a spacer salt is present, the new prepeak feature in the structure function (*S*(*q*)) is reminiscent of what is seen for ionic liquids^44−56^ where it is associated with polar–apolar alternation (i.e., polar networks spaced by apolar domains). In the case of LaCl_3_–NaCl mixtures, it is NaCl that plays the role of the less polar spacer domain. At lower concentrations of La^3+^, it is expected that Cl^–^ will be found in significantly different environments, as a counterion of Na^+^ or in complex with La^3+^. This will result in heterogeneity of Cl^–^ basicity, which is akin to the type of energetic heterogeneity often observed for ionic liquids.^57,58^ The Experimental Section S1, including Tables S1–S6, eqs S1–S14, and Figures S1–S3, provides all necessary methodological details for this article.

## Results and Discussion

**Figure 1:**
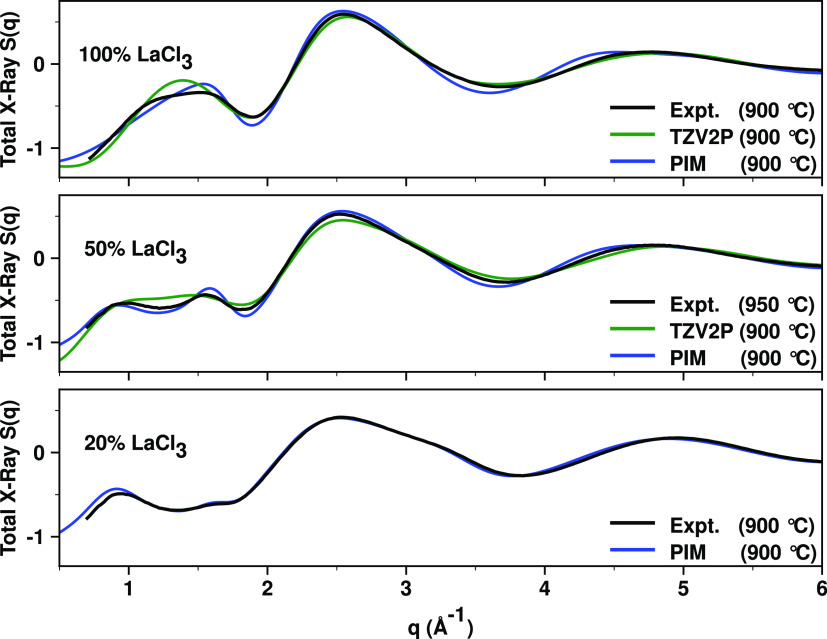
Comparison between experimental and simulated X-ray *S*(*q*) for LaCl_3_ and LaCl_3_–NaCl mixtures. Ab initio molecular dynamics (AIMD) results are shown for the TZV2P basis set; the DZVP basis set gives practically identical results as can be gleaned from Figure S4. Experimental and computational data on the full experimental *q*-range are shown in Figure S5.

**Figure 2:**
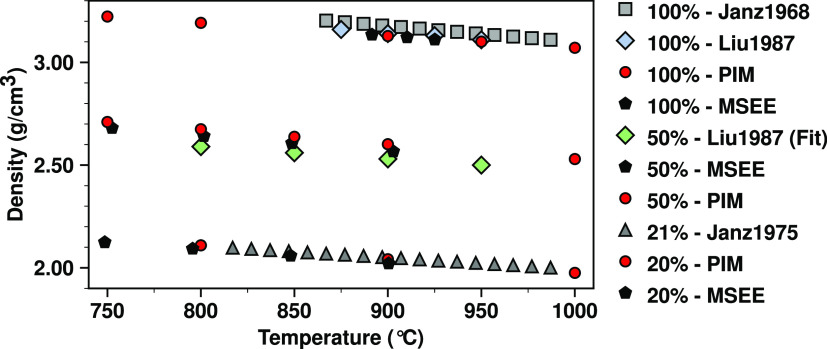
Average PIM simulated densities and experimentally measured densities of LaCl_3_ and various LaCl_3_–NaCl mixtures; PIM simulations from this work (red circles), Janz1968 expt.^59^ (gray squares), Janz1975 expt.^60^ (dark gray triangles), Liu1987 expt.^61^ (light blue diamonds), and our linear interpolation of values from Liu1987 expt.^61^ for 50% LaCl_3_ (green diamonds). To achieve a better fit, for the 50% mixture, we dropped the lowest two LaCl_3_ concentrations. Our own density measurements discussed in Section S1.3 are labeled MSEE (black pentagons); actual density values and errors for these are reported in Table S2. Simulations of LaCl_3_ for the lowest two temperatures are below the known melting point of the neat salt.

Our discussion starts with a comparison of X-ray *S*(*q*) measurements against our different simulation techniques in Figure 1. The goal is to derive conclusive real- and reciprocal-space information about the specific ionic correlations giving rise to the experimental peaks as a function of La^3+^ concentration, and for that, we must first establish that simulations provide an accurate representation of the experimental results. Figure 2 provides computational data that can be contrasted against density measurements from this work and other sources, in particular new lower-temperature measurements for the 50 and 20% LaCl_3_ mixtures that were unavailable in the literature.

In the case of neat LaCl_3_, neither our first-principles MD simulations nor the PIM provides a perfect match to the scattering experiments in Figure 1; yet, it is clear that all simulation techniques capture at the correct *q* values the different features of *S*(*q*). For LaCl_3_, the AIMD results appear more accurate, at least at higher *q* values, but as the concentration of LaCl_3_ lowers to 50 and 20%, it is clear that the PIM produces excellent results in comparison to experiments; this is likely due to the robust parameterization by Ishii et al.^62^ of force field parameters for the monovalent salts. One important point that we will address in this article is the interpretation of a prepeak or first sharp diffraction peak (FSDP) below 1 Å^–1^ that is absent for neat LaCl_3_ but develops upon dilution. This peak is different in nature from the reported prepeak observed for neat LaCl_3_ in neutron scattering experiments.

### Physical Interpretation of Structure in the LaCl_3_ and LaCl_3_–NaCl Melts

**Figure 3:**
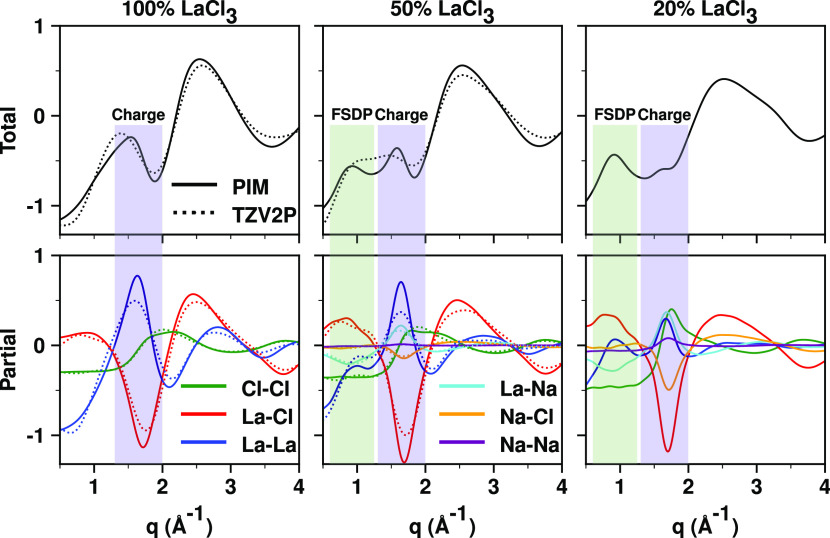
Total X-ray *S*(*q*) and partial subcomponents at 900 °C from PIM and first-principles simulations using the TZV2P basis set.

We see from Figure 3(left) that when considering pure LaCl_3_, the La–La partial subcomponent of the X-ray *S*(*q*) shows no distinct prepeak separated from the usual charge alternation peak. We conclude from this that, if there really are two distinct La–La structural correlation length scales in the neat melt, one giving rise to charge alternation behavior and another to some sort of intermediate-range order, the difference in real-space distances between La^3+^ ions involved in each of these two has to be quite small. Note that this is different from the situation that is observed when diluting LaCl_3_ with NaCl, where two separate peaks develop at a low *q*, one above 1.5 Å^–1^ and one below 1 Å^–1^ in Figure 3(middle and right, respectively) as well as in the experimental *S*(*q*) shown in Figure 1. We will expand on this after interpreting the behavior of partial *S*(*q*) subcomponents in neat LaCl_3_ salt below 3 Å^–1^. We focus on the below 3 Å^–1^ reciprocal-space regime, because in Figure S6, we see that there are no structural correlations below 2π/3 ∼ 2.09 Å in any of the pair distribution functions.

For neat LaCl_3_ at low *q* values, the first three features we observe in Figure 3(bottom left) are two peaks (blue and green lines) and one antipeak (red line) below 2 Å^–1^. This pattern of two peaks and one antipeak is the hallmark of charge alternation for all ionic liquids and all molten salts. For many salts and ionic liquids, the two peaks and the antipeak occur at very similar *q* value, but note that for LaCl_3_, this is not the case due to the large size mismatch of the ions. In the most simplistic terms, one can think of the aforementioned peaks as being associated with the typical real-space distance between ions that are spaced by a counterion. Instead, the red La–Cl antipeak is associated with the typical real-space distance from a La^3+^ ion where we do not expect to see a Cl^–^ ion (because this is where another La^3+^ normally would be); alternatively, the red antipeak can be interpreted as associated with the typical real-space distance from a Cl^–^ ion where we do not expect to see a La^3+^ ion. Note that the Cl–Cl peak is broad and extends beyond the charge alternation regime. This is because multiple Cl^–^ ions coordinate with La^3+^ in charge alternation networks and these Cl^–^ ions are necessarily close. In other words, for Cl^–^ in LaCl_3_, the charge alternation peak and the adjacency peak overlap forming a single broad feature that extends from ∼1.5 to >2.5 Å^–1^. Note also the adjacency peak (red line at ∼2.5 Å^–1^) for La–Cl correlations. This is associated with La^3+^ ions and Cl^–^ ions that are in close contact; there is no adjacency peak for La–La correlations simply because Coulomb repulsions prevent La^3+^ ions from ever being in an adjacency situation (see La–La pair distribution function in Figure S6).

This brings us to the question of what the origin is for the prepeak that develops upon dilution of La^3+^ in LaCl_3_–NaCl mixtures. To explain this, we need to fall back on the concept of same-type vs opposite-type structural correlations that we have developed in multiple publications.^46,47,53,63^ In the charge alternation regime (see blue and cyan lines in Figure 3(bottom middle and right) at *q* ∼ 1.65 Å^–1^), the La–La and La–Na peaks correspond to “same-type” structural correlations; these peaks are associated with positive ions that are spaced by counterions, i.e., La–Cl–La and La–Cl–Na charge alternation structural motifs. The opposite is true in the prepeak region around 0.9 Å^–1^ (see cyan line antipeak in Figure 3(bottom right) corresponding to the 20% LaCl_3_ mixture), where La^3+^ and Na^+^ ions behave as “opposite-type” species. The meaning of this is that NaCl is acting as a spacer in between La^3+^ ions. These La^3+^ ions can either be part of La^3+^ networks or be isolated complexes. The next section shows this schematically in real space.

**Figure 4:**
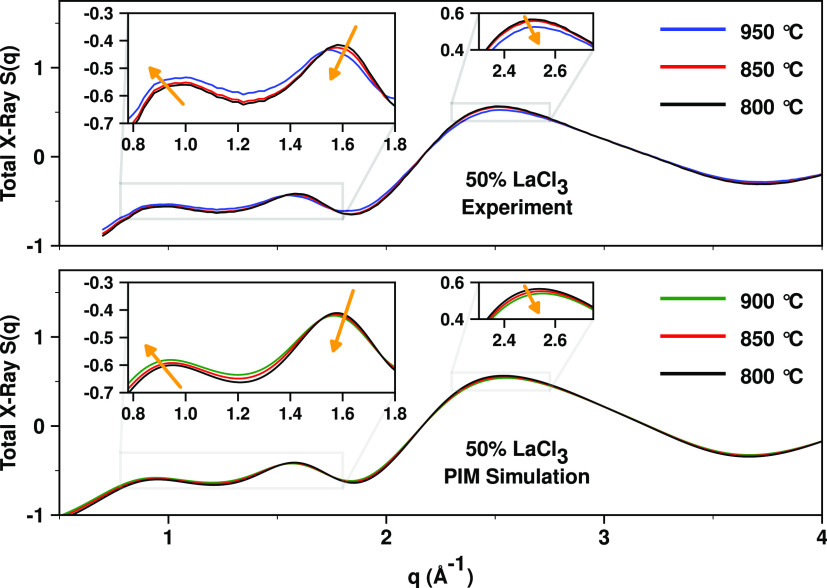
Total X-ray *S*(*q*) for 50:50 LaCl_3_–NaCl at various temperatures from experimental measurements (top) and calculated from PIM simulations (bottom).

A characteristic of first sharp diffraction peaks for many ionic liquids and molten salt systems is that these tend to have anomalous temperature behavior.^64,65^ In other words, although the charge alternation peak and the adjacency peak diminish in intensity with increasing temperature (this is typical Debye–Waller behavior), the prepeak does the opposite, increasing in intensity at a high *T*. Our experiments and simulations shown in Figure 4 confirm that this is the case for the LaCl_3_–NaCl system.

### Real-Space Interpretation of the Prepeak and the Concept of a Spacer Salt

**Figure 5:**
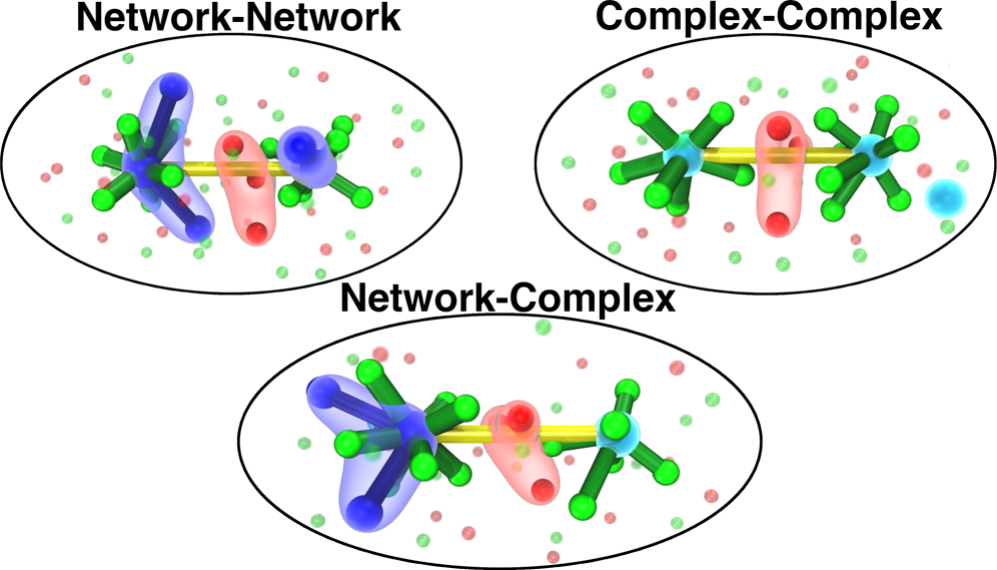
Fragments of snapshots from an equilibrated PIM simulation trajectory of 20% LaCl_3_–80% NaCl at 900 °C showing La–La pair interactions that significantly contribute to the prepeak depicted with connecting yellow lines. Local configurations of “networked” La^3+^ ions with *r*_La–La_ ≤ 4.9 Å (blue) and “free” La^3+^ complexes (cyan) are shown including their nearest-neighboring Cl^–^ ions with *r*_La–Cl_ ≤ 4.2 Å (green). To highlight NaCl as the “spacer salt”, Na^+^ ions (red) that are within 4.0 Å of the midpoint between the two La^3+^ ions are also highlighted. Further ions are shown as smaller transparent spheres for clarity.

A more schematic view of the prepeak can be achieved if the following question is asked: for a simulation frame, what specific La–La pair interactions contribute the most to the prepeak in the 20% LaCl_3_–NaCl mixture while at the same time approximately matching the Bragg condition *d* ∼ 2π/*q*_prepeak_? We can address this question if instead of taking Fourier transforms of the PDFs to compute *S*(*q*), we instead analyze this function as a sum over pairs of ions in reciprocal space.^55,66^Figure 5 provides a simple answer; in all cases, it is La^3+^ ions separated from other La^3+^ ions by a single layer of the spacer salt. As for the type of La^3+^ involved, these could be in a chloride-decorated La^3+^ network, in disconnected or off-network complexes or in a mixture of both. Figure 5(left, middle, and right) shows the typical case of two distinct chloride-decorated La^3+^ networks spaced by NaCl, a network separated from a complex via the spacer salt, and two distinct La^3+^ complexes spaced by NaCl. Note that the spacer salt can be described almost as a monolayer; this is the only way the Bragg condition can be approximately fulfilled.^a^

**Figure 6:**
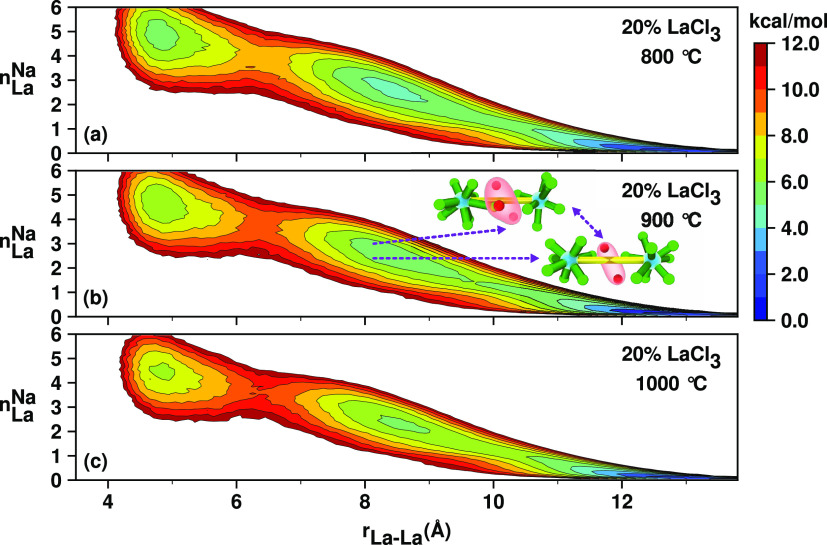
2D free energy surfaces as a function of (1) number of shared Na^+^ ions between two La^3+^ ions (*n*_La_^Na^) and (2) their separation distance (*r*_La–La_). The minima in between 8 and 9 Å, which coincide with the prepeak in reciprocal space, show that two or three Na^+^ ions act as spacers between La^3+^ ions matching the discussion associated with Figure 5.

Two-dimensional (2D) free energy plots discussed extensively in subsequent sections can be crucial to separate correlations that are present in the same distance regime of the pair distribution function. Figure 6 shows that in a “prepeak configuration” where two La^3+^ ions are spaced between 8 and 9 Å, there are normally 2–3 Na^+^ ions in between these, matching the results obtained by sorting contributions of ion pairs in the direct sum method to calculate *S*(*q*).

### Most Intense Prepeak Appears in the Absence of La^3+^ Networks

**Figure 7:**
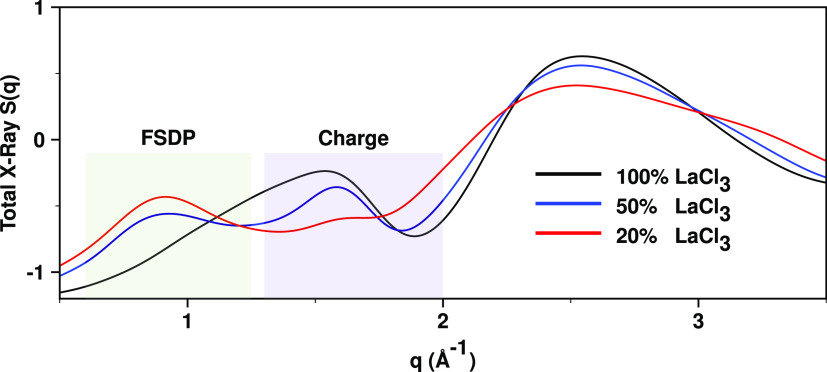
*S*(*q*) as a function of LaCl_3_ concentration at 900 °C from PIM simulations.

Figure 7 depicts the changes in *S*(*q*) as a function of LaCl_3_ concentration. It is apparent from the figure that the charge alternation peak intensity decreases as we decrease the LaCl_3_ concentration, but the opposite occurs to the prepeak which increases in intensity with decreasing LaCl_3_ concentration. Prepeaks are often intuitively linked to the formation of “structures”, and since the partial La^3+^–La^3+^ subcomponent of *S*(*q*) is a strong contributor to the prepeak, one could erroneously arrive at the conclusion that larger La^3+^ networks correspond to more prominent prepeaks.

**Figure 8:**
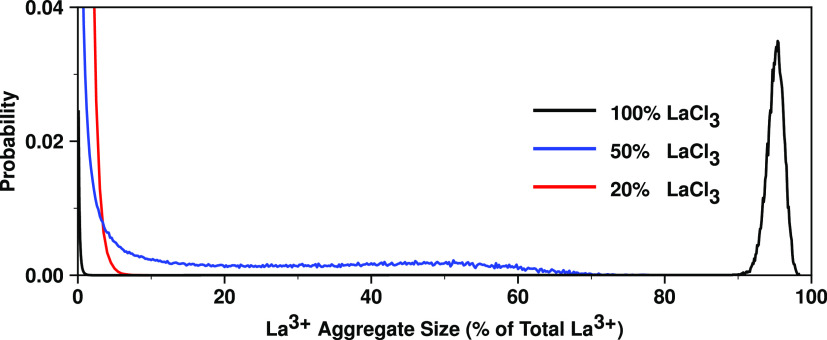
AGGREGATES^67^ network size analysis for La^3+^–La^3+^ correlations as a function of concentration at 900 °C using the PIM. A cutoff distance of 4.9 Å between two La^3+^ ions is used to define connectivity. This value, albeit somewhat arbitrary, is consistent with the onset of the charge alternation peak in the partial structure function and also captures a significant portion of the first La–La peak of the RDF in Figure S6 for the 50 and 20% mixtures.

In fact, the opposite is true; the reason for the increase in the prepeak is likely the disappearance of Cl^–^-decorated La^3+^ networks, which can be discussed in the context of network size analysis using the AGGREGATES program written by Bernardes.^67^Figure 8 shows that for neat LaCl_3_ where there is no first sharp diffraction peak, essentially the whole liquid is part of the same network. As we increase the concentration of the spacer salt, the fraction of La^3+^ ions that are in-network decreases, the networks become shorter, and the prepeak increases in Figure 7. This means that many configurations such as those in Figure 5(right) are the ones that are important as opposed to the networked configurations in Figure 5(left and middle). In other words, entropy results in more across-complex interactions increasing the intensity of the prepeak.

### Coordination Numbers and Coordination Free Energetics

**Figure 9:**
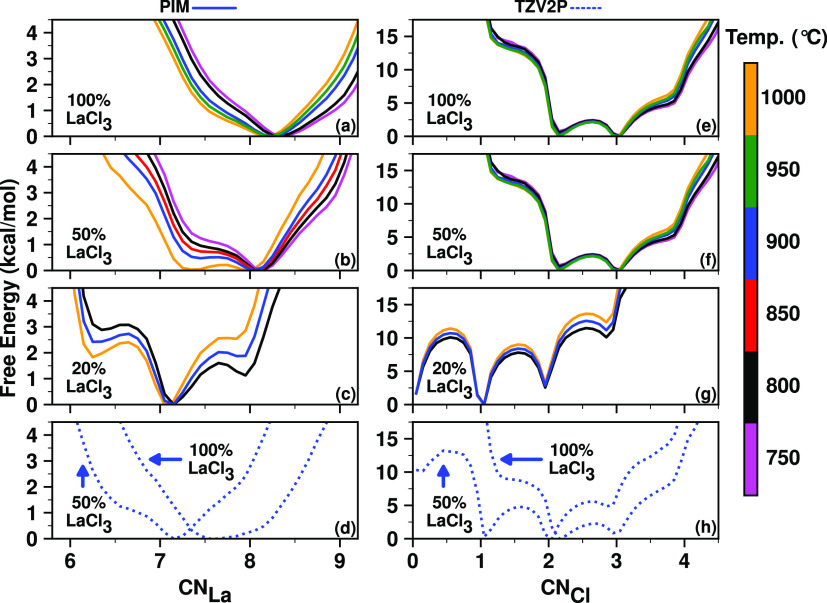
Free energy profiles for CN_La_ (a–d) and CN_Cl_ (e–h) reflecting the effects of LaCl_3_ concentration (mol %) on the La–Cl coordination environment revealed by the PIM (top three rows; all solid lines at different temperatures indicated by different colors) and TZV2P AIMD (bottom row; dotted blue lines at 900 °C) simulations; results with the DZVP basis set (not shown) are very similar to those with the TZV2P basis set.

Thus far, our discussion has been based on the idea of networks and complexes, but we have not been quantitative when discussing coordination in these structural motifs. The reason for this is that at the temperature where these systems are in the molten state, it is often simply not appropriate to think of a fixed coordination number. The local La–Cl coordination environment and its fluctuations/reorganization in the high-temperature melt can be best understood in terms of different metastable states of Cl^–^ ions around a La^3+^ ion and vice versa. The chloride coordination number of La^3+^, namely, CN_La_, is found to be sensitive to both temperature and the concentration of La^3+^ ions as can be gleaned from the free energy profiles shown in Figure 9a–d.

For the pure LaCl_3_ melt, the PIM simulations (see Figure 9) reveal that while the most-likely CN_La_ is ∼8 across all temperatures, the higher (≥900 °C) and lower (≤800 °C) temperatures, respectively, allow frequent but transient escapes to lower and higher CN_La_ values (e.g., ∼7 to 7.5 and ∼9, respectively). These two are shallow free energy minima (without any barriers separating them from the global minimum CN_La_ ∼ 8) that are easily accessible at the high temperatures of our studies. For the 50:50% mixture melt with NaCl examined in the PIM simulation (Figure 9b), CN_La_ ∼ 7 and CN_La_ ∼ 8 became equally probable and dominating at the highest temperature (1000 °C). As the temperature decreases, this mixture melt shows increasing preference for CN_La_ ∼ 8 compared to CN_La_ ∼ 7. The PIM simulations show a drastic change in the coordination environment (and concomitant free energy profile) for the 20:80% mixture melt with NaCl at 900 °C, wherein CN_La_ ∼ 7 becomes the dominating state followed by the less probable CN_La_ ∼ 8 and CN_La_ ∼ 6. At other temperatures, the trend is similar to that for the 50% mixture in that preference for the lower CN_La_ ∼ 6 increases at high temperatures at the expense of the CN_La_ ∼ 8 state, and the opposite is true when the temperature is decreased. It is evidently clear from Figure 9a–c that increasing the temperature or decreasing the concentration of La^3+^ both tend to reduce its chloride coordination number. While the AIMD results (Figure 9d) indicate the same effects as a function of La^3+^ concentration, lower coordination numbers are more sampled compared to the PIM results. For example, the AIMD free energy profile has a broad minimum at CN_La_ ∼ 7.5–8 for the pure LaCl_3_ melt and the most-likely CN_La_ is ∼7 for the 50:50% mixture.

The effect of temperature on the lanthanum coordination of a Cl^–^ ion is rather negligible as found from the PIM simulations (Figure 9e–g), but the effect of decreasing the LaCl_3_ mol % is significant and analogous to what is observed for the chloride coordination of a La^3+^ ion. In the pure LaCl_3_ melt, either two or three La^3+^ ions coordinate with a Cl^–^ ion with similar probability, whereas in the 50:50% mixture, the CN_Cl_ ∼ 1 state accompanies the CN_Cl_ ∼ 2 and CN_Cl_ ∼ 3 states alongside the less-frequently occurring CN_Cl_ ∼ 0 state. The reduction of the most probable values of CN_Cl_ is more prominent for the 20:80% mixture (Figure 9g), wherein the most-likely CN_Cl_ is ∼1 and the lesser-likely CN_Cl_ ∼ 0 and CN_Cl_ ∼ 2 states are almost equally probable. The AIMD results (Figure 9h) qualitatively agree with these findings from the PIM simulations. As will be discussed later, the heterogeneity and relative metastability of these CN states encoded in the free energy profiles will be useful in interpreting the Raman spectra computed from the AIMD trajectories. For convenience and to help in the interpretation of Raman spectra, the exact populations of different CN states obtained from simulation are given in Table S7.

### Free Energy of the Liquid Motifs and Relation to *S*(*q*)

**Figure 10:**
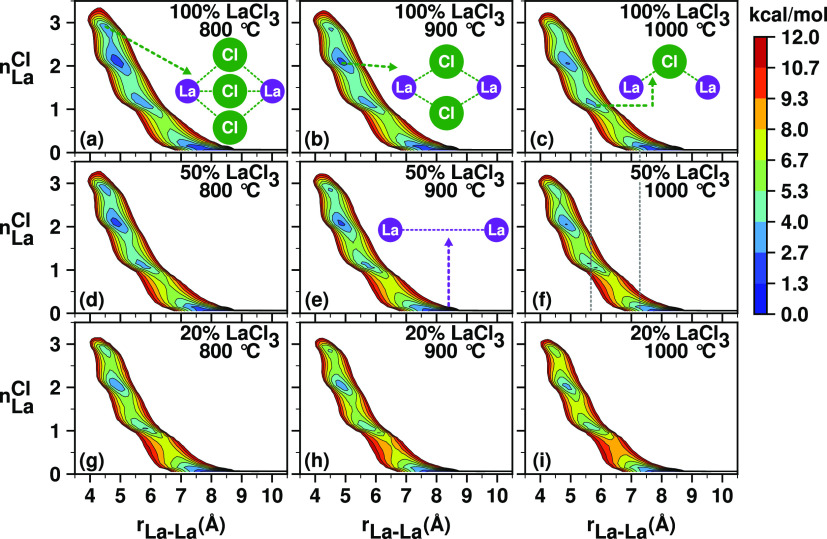
2D free energies from the PIM simulations as a function of (1) number of shared Cl^–^ ions between two La^3+^ ions (*n*_La_^Cl^) and (2) their separation distance (*r*_La–La_). The effects of increasing temperature (left to right) and decreasing La^3+^ concentration (top to bottom) are similar: they diminish −La–(*n*_La_^Cl^)Cl–La– along-chain correlations.

**Figure 11:**
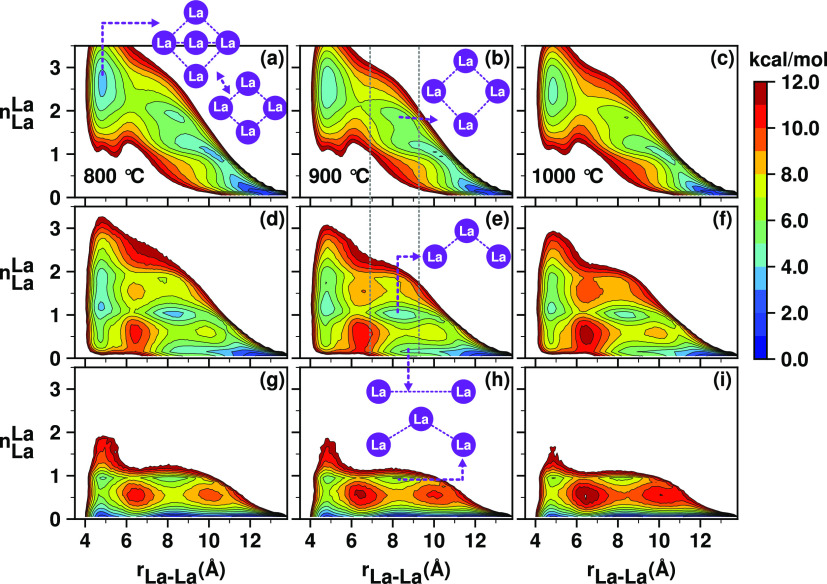
2D Free energy surfaces, *W*(*r*_La–La_, *n*_La_^La^), obtained from the PIM simulations, highlighting the temperature and concentration effects on the formation of La^3+^ liquid structural motifs; 100% LaCl_3_ (a–c), 50% LaCl_3_/50% NaCl (d–f), and 20% LaCl_3_/80% NaCl (g–i).

While one-dimensional (1D) free energy surfaces in Figure 9 provide an in-depth understanding of the heterogeneity and metastability of the local La–Cl coordination, it is important to understand that such coordination occurs in the context of larger liquid motifs. In other words, these are not necessarily the coordination numbers of isolated metal complexes but instead possibly of chains, networks, and loops. The 2D surfaces defined in eqs S12 and S13 and illustrated in Figures 10 and 11 depict how pairs of La^3+^ ions interact through Cl^–^ mediation forming specific local structural motifs.

For the pure LaCl_3_ melt, the free energy surfaces *W*(*r*_La–La_,*n*_La_^Cl^) in Figure 10a–c suggest that when two La^3+^ ions are at close range (*r*_La–La_ ≤ 6.4 Å, see first La–La peak range in the RDF shown in Figure S6), their interaction is mediated by sharing one, two, or three Cl^–^ ions, but the two-shared Cl^–^ state (also known as the edge-sharing state) is the most probable. Figure 11a shows that rapidly interconverting locally pentameric and tetrameric configurations of La^3+^ ions can form as parts of extended Cl^–^-decorated −La–La– networks. Specifically, *W*(*r*_La–La_,*n*_La_^La^) in Figure 11a–c shows that at *r*_La–La_ ≤ 6.4 Å, a La^3+^ ion is surrounded by two or three other lanthanum ions.

Note how, as temperature increases, the free energies associated with these local motifs at *r*_La–La_ ≤ 6.4 Å (Figure 11a–c) become shallow. Because these are the minima corresponding to the motifs giving rise to the La–Cl–La in-network correlations, the observation can be linked to the drop in *S*(*q*) intensity with temperature at *q* corresponding to the charge alternation peak (i.e., simple Debye–Waller behavior). As a function of decreasing La^3+^ concentration and in the same range *r*_La–La_ ≤ 6.4 Å, there is a decrease in *n*_La_^La^ corresponding to the global free energy minimum where the position of the minimum shifts from *n*_La_^La^ being in the range 2–3 to a value of 1 at 50% LaCl_3_ and 0 at 20%. This is consistent with a lower-intensity charge alternation peak with diminishing La^3+^ concentration in Figure 3 (blue line around 1.6 Å^–1^); see also Figure 7 in the same *q* regime.

To explore the correlations in *S*(*q*) that are at lower *q* (longer distance than typical La–Cl–La charge alternation), we examine pairs of lanthanum ions with separation distances ≥ 6.4 Å. From all subplots in Figure 10, we see that geometry simply does not allow sharing of Cl^–^ ions by pairs of La^3+^ ions at these longer distances. La^3+^ ions in the important range 6.4 Å ≤ *r*_La–La_ ≤ 10 Å, can be in-network but too far apart to share counterions, or simply not in the same network. Interestingly, it is notable from the free energy surfaces in Figure 11 that even longer La–La structural correlations exist at *r*_La–La_ ≈ 11–13 Å even though these may not be easily resolved in *S*(*q*).

For the pure LaCl_3_ melt, Figure 11a–c indicates that in the intermediate range 6.4 Å ≤ *r*_La–La_ ≤ 10 Å, there are always one or two La^3+^ ions between two other La^3+^ ions. In other words, La^3+^ ions are always networked and there is no prominent peak in *S*(*q*) below 1 Å^–1^. Instead, when LaCl_3_ is mixed with the spacer salt NaCl at 50%, for 6.4 Å ≤ *r*_La–La_ ≤ 10 Å in Figure 11d–f, we can clearly distinguish a solvent-separated La–La state and an in-network state; the first one identified at *n*_La_^La^ = 0 and the second one at *n*_La_^La^ = 1. The solvent-separated La–La state becomes much more prominent for the 20% LaCl_3_ mol fraction, as established by the deeper minimum (see, for example, Figure 11h; at *n*_La_^La^ = 0 and *r*_La–La_ ≈ 8–9 Å compared with the 50% LaCl_3_ mol fraction in Figure 11e; at *n*_La_^La^ = 0 and *r*_La–La_ ≈ 8–9 Å). The appearance of this solvent-separated state with dilution can be directly associated with the FSDP at *q* below 1 Å^–1^. In other words, in this case, less La^3+^ networked structure causes an increase in the FSDP, not a decrease.

### Must We Invoke Octahedral Coordination to Interpret the Raman Signal?

Previous analysis of the experimental Raman spectra had suggested a sixfold coordination of LaCl_3_ in all compositions, including pure LaCl_3_.^68,69^ The isomorphous structural assignment in all compositions was supported by the similarity of the Raman spectra across the composition range through a series of mixtures from dilute solutions to pure LaCl_3_ and the other lanthanide halides.^70^ However, this interpretation disagrees with higher coordination numbers (7–8) in pure LaCl_3_ obtained from previous atomistic simulations,^4,41^ X-ray^33,41^ and neutron diffraction,^38^ and EXAFS^33^ studies. It also disagrees with our molecular simulations in which an ensemble of coordination environments is observed for La^3+^ with average coordination number that shifts to larger values as the LaCl_3_ concentration increases; Figure 9 and Table S7 show that the average coordination number of La^3+^ in neat LaCl_3_ is ∼8.2 for the PIM and ∼7.7 for AIMD.

To resolve this controversy, we have applied our recently developed methodology to simulate Raman spectra based on the time-dependent polarizability tensor of the full periodic simulation cell obtained from the AIMD trajectory and compared the simulated spectra for pure molten LaCl_3_ with published results^68^ and that of the binary 50:50% mixture of LaCl_3_ and NaCl with new measurements performed in the current work.

**Figure 12:**
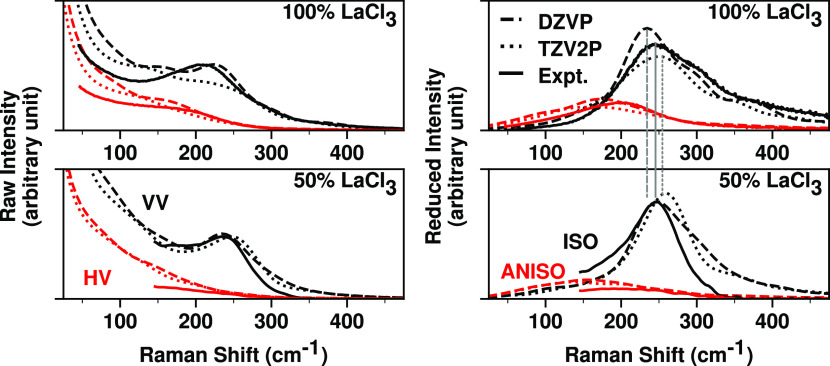
Raman spectra calculated from AIMD simulations vs experimental measurements for pure LaCl_3_ by Zissi et al.^68^ (top) and new experimental measurements for a 50:50% mixture of LaCl_3_–NaCl (bottom). Experimental measurements are shown as solid curves, while dashed and dotted curves are used for the computed data from the AIMD simulations using DZVP and TZV2P basis sets, respectively. Raw Raman spectra are shown in the left panels, including parallel (VV—black lines) and perpendicular (HV—red lines) orientations, while reduced Raman spectra are shown in the right panels; isotropic (ISO—black lines) and anisotropic (ANISO—red lines). We use vertical drop lines to indicate the approximate position of the maximum intensity in the reduced isotropic Raman spectra of pure LaCl_3_.

As the raw Raman spectra are rather featureless, the spectra in the reduced representation (eq S14) are more informative in analyzing typical energy excitations for molten salts below 1000 cm^–1^. The reduced Raman spectra for isotropic (ISO) and anisotropic (ANISO) intensities are shown in Figure 12(right), together with the experimental spectra^68^ of LaCl_3_ and the 50:50% mixture of LaCl_3_ and NaCl. The main peaks in the isotropic spectra (P1) due to the symmetric stretching of the coordinated La^3+^ complexes closely match the bands in the experimental spectra. Experimentally, there is a slight shift (∼4 cm^–1^) in the position of the main band to lower energy and substantial broadening with increasing the mole fraction of LaCl_3_ from 50 to 100%. Simulation results from different basis sets match well the observed broadening and just like the experiment show a very small shift in the same direction or no shift. This settles the disagreement in the previous interpretation of the Raman data since it is possible to accurately predict the isotropic Raman spectra from the AIMD simulations in which the range of coordination numbers is, as Table S7 indicates, on the range 6–9 with population shifts depending on the La^3+^ concentration. In other words, significant changes in the coordination number with increasing LaCl_3_ mole fraction produce broadening and only minor changes in the P1 band frequency.

Interestingly, for the LaCl_3_–CsCl system that has been previously studied on a wider range of compositions,^68^ the frequency of the P1 band first increases from 241 cm^–1^ for 5 mol % LaCl_3_ to 261 cm^–1^ for 68 mol % LaCl_3_ and then decreases with increasing concentration. Such spectral changes are likely to be the result of two competing factors that influence the P1 band energy in opposite ways. The overlap of the coordination polyhedra strengthens the La–Cl bond involving terminal chloride anions, while the increase in the number of chloride neighbors weakens the La–Cl bond, significantly complicating structural assignment from the experimental Raman data for concentrated melts without the help of simulation data. A full analysis and comparison of the ANISO band is beyond the scope of this study as it would require experimental mixture results at lower energies. In a future study, the relative intensity at different frequencies for Raman bands (such as the D1 and D2 bands reported in ref (68) for CsCl) could be used as a very stringent test for basis sets and density functional theory (DFT) flavors.

## Conclusions

In this article, we have attempted to provide a fully consistent experimental and computational interpretation of the structure of LaCl_3_ and its mixtures with NaCl from a multiprong approach using different experimental measurements and computational techniques. Although many questions are answered, a few points are most important. (1) The coordination structure of La^3+^ and likely other lanthanides and actinides is not only concentration-dependent but also at each concentration, multiple well-defined coordination states exist with populations that change with temperature. (2) A prepeak in scattering appears upon mixing LaCl_3_ with NaCl that is due to lower-charge solvent (NaCl)-separated correlations between Cl^–^-decorated La^3+^ complexes or networks. This peak is absent when considering each of the two pure component salts. (3) The claim of an apparent controversy between Raman results that prescribe a specific coordination number (sixfold) for La^3+^ and other experiments that contradict this, implying the coordination number is larger, should be abandoned as it is clear that simulations predicting a larger coordination number for La^3+^ (in fact an ensemble of coordination numbers) reproduce the Raman spectra well.

The work we presented here goes a long way in correlating reciprocal-space features in *S*(*q*) with specific real-space three-dimensional structural motifs. These motifs also correspond to minima in 2D free energy landscapes; we hope that our work identifying and cataloging these will be useful to those trying to assign similar structural patterns for other lanthanides and actinides in the molten state. The work discusses the effect of concentration on the size of Cl^–^-decorated La^3+^ aggregates and connections are made between the role of lower-charge “spacer solvents” and what is commonly found for ionic liquids, where a lower-charge tail domain occurs that also gives rise to a first sharp diffraction peak. Just like for LaCl_3_–NaCl mixtures, for ILs, the prepeak (but not other peaks) often displays anti-Debye–Waller temperature behavior. The article also provides new measurements of the physical properties for LaCl_3_ and its mixtures in regimes not previously studied, which we hope will be useful to the community in general.

## Supporting Information Available

Experimental section describing scattering and Raman experiments, density measurements, first principles, and polarizable ion model simulations as well as calculated properties including the X-ray *S*(*q*) and its subcomponents, ionic coordination numbers, free energies, and Raman spectra; comparison of TZV2P and DZVP total X-ray structure functions; extended-range experimental and simulated total X-ray structure functions; simulated pair distribution functions; and table of computed coordination numbers (PDF)

## Notes

Data sets for this article are made available within 30 days of the official acceptance date of this article by the journal in the Zenodo repository under the Digital Object Identifier (DOI): 10.5281/zenodo.7093225.

## Footnotes

a *d* is often slightly larger than 2π/*q*.
