# Supplementary material for: Complete Description of the LaCl3–NaCl Melt Structure and the Concept of a Spacer Salt That Causes Structural Heterogeneity
Source: J Am Chem Soc. 2022 Nov 15;144(47):21751–62. doi: 10.1021/jacs.2c09987 (PMC9716573; doi:10.1021/jacs.2c09987)
Supplement: Supplementary file 1 — ja2c09987_si_001.pdf [file ja2c09987_si_001.pdf]

Supporting Information for: Complete Description of the $LaCl_3$-NaCl Melt Structure and the Concept of a Spacer Salt that Causes Structural Heterogeneity

Matthew S. Emerson,[†] Shobha Sharma,[†] Santanu Roy,[*,‡] Vyacheslav S. Bryantsev,[*,‡] Alexander S. Ivanov,[*,‡] Ruchi Gakhar,[*,¶] Michael E. Woods,[¶] Leighanne C. Gallington,[§] Sheng Dai,[‡,∥] Dmitry S. Maltsev,[‡] and Claudio J. Margulis[*,†]

†*Department of Chemistry, The University of Iowa, Iowa City, IA 52242, United States*

‡*Chemical Sciences Division, Oak Ridge National Laboratory, Oak Ridge, TN 37831, United States*

¶*Pyrochemistry and Molten Salt Systems Department, Idaho National Laboratory, Idaho Falls, ID 83415, United States*

§*X-ray Science Division, Advanced Photon Source, Argonne National Laboratory, Lemont, IL 60439, United States*

∥*Department of Chemistry, University of Tennessee, Knoxville, TN 37996, United States*

E-mail: roys@ornl.gov; bryantsevv@ornl.gov; ivanova@ornl.gov; ruchi.gakhar@inl.gov; claudio-margulis@uiowa.edu

Contents

S1 Experimental Section **S3**

S1.1 Syncrotron X-Ray Scattering Experiments S3

S1.2 Raman Spectroscopy Experiments S5

S1.3 Density Measurements S5

S1.4 Ab-Initio Molecular Dynamics (AIMD) Simulations S8

S1.5 Polarizable Ion Model (PIM) Simulations S10

S1.6 Calculated Properties from Simulations S13

S1.6.1 Structure Functions S13

S1.6.2 Coordination Numbers S13

S1.6.3 2D Free Energy Calculations S14

S1.6.4 Raman Spectroscopy S15

S2 Supplemental Figures and Tables **S17**

References **S20**

S1 Experimental Section

Table S1 provides a list of concentrations, temperatures and techniques that are relevant to our different studies.

Table S1: Table of Systems and Applied Techniques

Salt	[$LaCl_3$] (Mol%)	Temp. (°C)	Sim.		Expt.	
			AIMD	PIM	X-ray	Raman
$LaCl_3$	100%	750		✓		
$LaCl_3$	100%	800		✓		
$LaCl_3$	100%	900	✓	✓	✓	
$LaCl_3$	100%	950		✓		
$LaCl_3$	100%	1000		✓		
$LaCl_3$-NaCl	50%	750		✓		
$LaCl_3$-NaCl	50%	800		✓	✓	
$LaCl_3$-NaCl	50%	850		✓	✓	
$LaCl_3$-NaCl	50%	900	✓	✓		✓
$LaCl_3$-NaCl	50%	950			✓	
$LaCl_3$-NaCl	50%	1000		✓		
$LaCl_3$-NaCl	20%	800		✓		
$LaCl_3$-NaCl	20%	900		✓	✓	
$LaCl_3$-NaCl	20%	1000		✓		

S1.1 Syncrotron X-Ray Scattering Experiments

Total X-ray scattering measurements for molten $LaCl_3$, $LaCl_3$-NaCl (20-80 mol%), and $LaCl_3$-NaCl (50-50 mol%) salts at different high temperatures were performed using the beamline 11-ID-B at the Advanced Photon Source (APS) with an incident X-ray wavelength of 0.2115 Å (energy = 58.6 keV). The diffracted X-rays were collected employing an amorphous silicon-based area detector (Perkin Elmer XRD1621) with 200×200 micron pixels. The detector was placed 160 mm behind the sample, giving an approximate accessible q-range of 0.6—15 $Å^{-1}$. Calibration of the precise sample to detector distance, detector tilt and rotation, and beam center was performed in the GSAS II program[1] using crystalline CeO_2 powder as the standard. Anhydrous $LaCl_3$ and NaCl salts in sealed quartz ampules (99.99% purity) were purchased from Aldrich-APL. The samples were prepared by mixing proper masses of $LaCl_3$ and NaCl in a glovebox, and then the salt mix-

tures were fused together at 800 °C under dynamic vacuum following our standard procedures reported in previous studies.[2–4] The obtained salt compositions were then crushed to fine powders, added to separate thin-walled quartz capillaries (1.5 mm O.D., 0.010 mm wall thickness, Charles Supper), and flame sealed near the top under vacuum. X-ray scattering data were collected for the molten samples in the furnace shown in Figure S1 originally designed by Chupas et al.[5] and for an empty quartz capillary at the same temperature to account for a background signal.

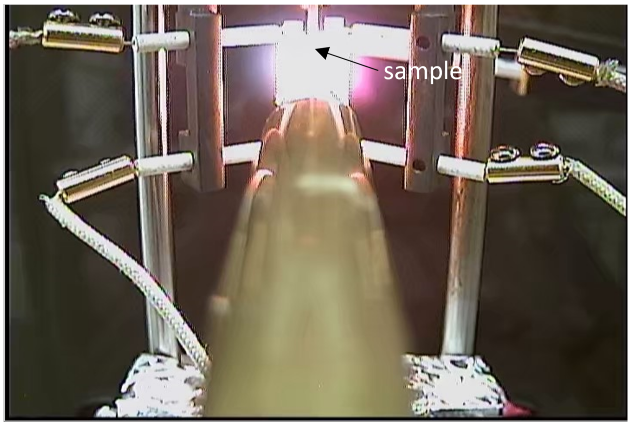

Figure S1: Syncrotron X-Ray Scattering Sample Holder/Furnace filled with 50% $LaCl_3$-NaCl mixture at 800 °C

GSAS II was then used to integrate the obtained 2D detector images to 1D diffraction patterns. Masks were applied to remove the beamstop and dead pixels from data integration. The PDFgetX2 software[6] was then used to subtract the background signal (empty quartz capillary) and to perform additional corrections (sample self-absorption, multiple scattering, and inelastic Compton scattering) following standard procedures.[7,8] The obtained data were subsequently normalized with respect to the average electron density given by the weighted sum of the corresponding ionic X-ray form factors,[9] yielding the total structure function, S(q):

$$S(q) = \frac{I_{coh}(q) - \sum_i x_i f_i^2(q)}{\left[\sum_i x_i f_i(q)\right]^2} \tag{S1}$$

where I_{coh} is the coherent scattering intensity, x_i and $f_i(q)$ are the molar fraction and q-dependent X-ray ionic form factor[9] of species i, respectively, and q denotes the magnitude of the scattering

vector ($q = 4\pi \sin(\theta)/\lambda$), where 2θ is the scattering angle, and λ is the incident X-ray wavelength.

S1.2 Raman Spectroscopy Experiments

The Raman spectrum of pure $LaCl_3$ at 900 °C was reported by Zissi et al.,[10] however, there is currently no experimental data available for the molten $LaCl_3$-NaCl mixtures to the best of our knowledge. In this work, we have performed high-temperature Raman measurements for the $LaCl_3$-NaCl (50-50 mol%) salt using a Horiba T64000 spectrometer. Two polarization configurations VV and HV (isotropic and anisotropic, respectively) were used for measuring the spectra. A diode laser of 532 nm was focused through a 50x objective of microscope. The power of the laser was 100 mW. The sample (≈50 mg) in an alumina crucible was measured using a Linkam heating stage TMS1100, which also allowed for an inert atmosphere during the experiment. This was done by connecting the stage to a nitrogen flow (15 mL/min). The Raman shift was measured from 150 to 700 cm^{-1} in the range that should cover the expected Raman signal of $LaCl_3$ molten salts.[10]

S1.3 Density Measurements

Density measurements were performed using the direct Archimedean method based on the buoyancy force exerted on a bobber submerged in molten salts; our results shown in Figure 2 are presented in comparison with our simulated data and, when available, with other experimental literature data. The density of the liquid salts can be calculated based on the equation:

$$\rho_{salts} = \frac{m_{argon} - w_{salts} + \frac{\pi D \gamma}{g}}{V_0 (1 + 3\alpha (T - T_0))} \tag{S2}$$

where m_{argon} is the measured mass of the bobber and wire suspended in argon, w_{salts} is the measured weight of the bobber and wire suspended in the salts, D is the diameter of the wire, γ is the surface tension of the salts, g is the gravity of Earth, α is the linear thermal coefficient of expansion of pure nickel, and T_0 is the reference temperature for V_0, the reference volume of the nickel bobber. The values of surface tension were calculated from reported values using the maximum

bubble pressure method and then creating linear interpolations and extrapolating to our temperature range.[11] The reported uncertainties in Table S2 follow standard error propagation based on instrument uncertainties and standard deviations of repeated measurements.

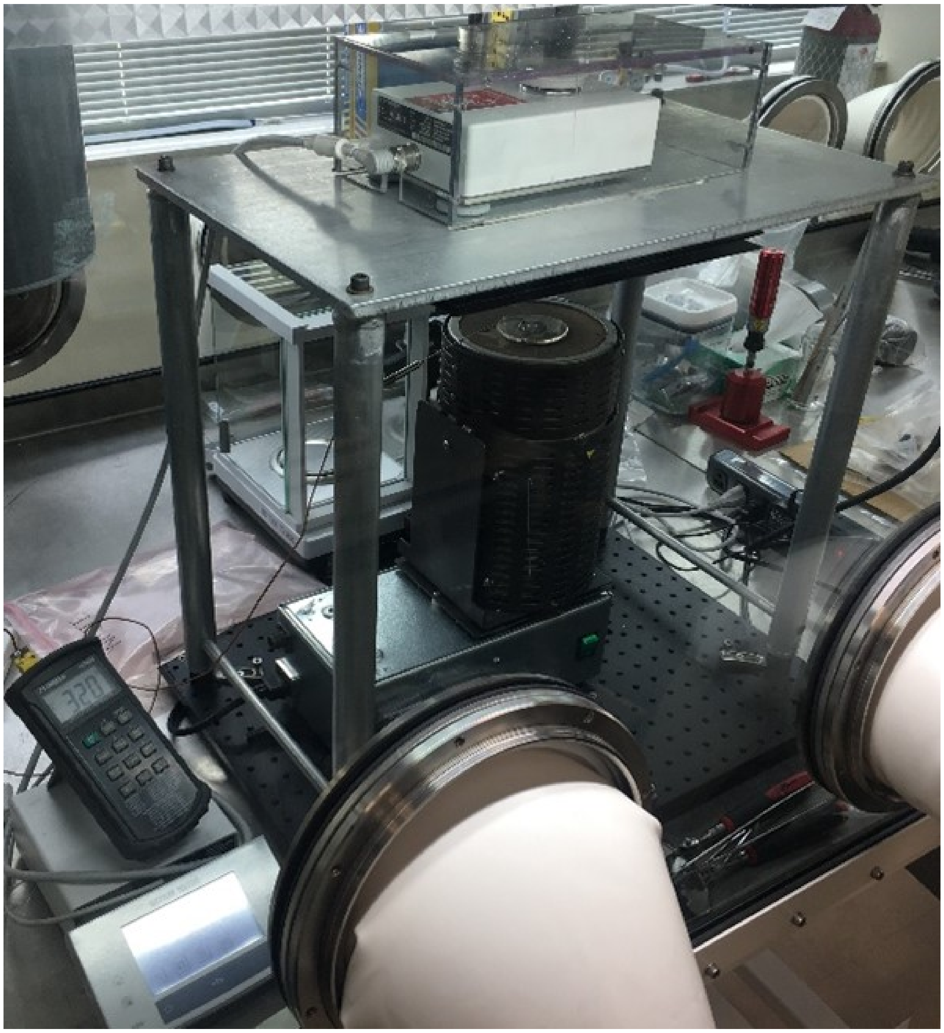

Figure S2: Experimental density measurement apparatus showing bottom-loading balance on the stand above the furnace inside an Argon atmosphere glovebox.

Table S2: List of density values and corresponding errors for the points labeled MSEE in Figure 2.

Salt	T (°C)	Density (g/cm^{-3})	Error (g/cm^{-3})
100% $LaCl_3$	891.5	3.1344	0.0334
	910.3	3.1218	0.0332
	925.1	3.1107	0.0331
50% $LaCl_3$	752.5	2.6805	0.0285
	801.6	2.6412	0.0281
	848.7	2.6068	0.0277
	902.9	2.5665	0.0273
20% $LaCl_3$	701.0	2.1547	0.0229
	748.6	2.1243	0.0226
	795.6	2.0927	0.0223
	848.0	2.0588	0.0219
	900.4	2.0213	0.0215

Figure S2 shows the custom designed setup including the furnace, stand, and a bottom-loading analytical balance housed inside an argon atmosphere glovebox operated at less than 2 ppm H_2O and O_2. A detailed description of the initial setup and testing can be found in Duemmler et al.[12] A Ni bobber (ESPI Metals, 99.98%) tied to 0.1 mm diameter tungsten wire (Alfa Aesar, 99.99%) was used for immersion and buoyancy measurements. Prior to measurements in molten salt, the volume of the Ni bobber was calculated at room temperature in fluids of well-defined densities, deionized water and ethanol. The average volume of $1.0393 \pm 0.0037 cm^3$ at 20 °C was obtained from these calculations. High-purity anhydrous NaCl and $LaCl_3$ salts were procured from Sigma-Aldrich and were used as received. The salt masses for mixtures were measured using a Mettler-Toledo balance (Model number TLE204E, tolerance 0.8 mg) located within the glovebox. Approximately 20 g of LaCl3 beads ($\geq$ 99.99% trace metal basis) were loaded into a glassy carbon crucible (GAZA2, 25 mL Sigradur cylindrical crucible, HTW Germany), which was priorly baked at 700°C for 4 hours to remove any adsorbed organic matter.

The mass of the bobber and wire was measured in argon using the bottom-loading analytical balance (Mettler Toledo WXSS204, tolerance 0.8 mg). The glassy carbon crucible loaded with salt was then introduced to the modified Ventura Electromelt furnace, and the salt sample was melted. The bobber was lowered into the molten salt, guided using a quartz lid. At each tempera-

ture set point, the sample was allowed to equilibrate until a steady temperature measurement was obtained by an Inconel-sheathed K-type thermocouple (OMEGA) inserted directly into the salts. An internal adjustment of the balance was performed before each set of mass measurements. After measurement with pure $LaCl_3$ the salt was allowed to cool down. The bobber was cleaned thoroughly with water and tied with a new tungsten wire once its weight measurement confirmed no mass loss or gain. The appropriate amount of NaCl beads (99.999% trace metal basis) were added to the crucible to achieve a 50%:50% molar ratio of $LaCl_3$ to NaCl. This 50%:50% mixture was then melted and the measurement procedure repeated to obtain measurements between 750-900°C, at increments of 50°C. For the measurements of 20 mol% $LaCl_3$ 80 mol% NaCl composition, a new mixture in a similarly prepared glassy carbon crucible was made and studied.

S1.4 Ab-Initio Molecular Dynamics (AIMD) Simulations

Simulation boxes pre-equilibrated at 900 °C using the PIM (*vide infra*) in the NPT ensemble with number of ions provided in Figure S3 and equilibration protocol described in Table S3 were used as input for AIMD simulations.

Table S3: PIM NPT Equilibration Procedure

Step Name	Temp. (K)	Time	τ_{Step} (fs)	$\tau_{Thermostat}$(fs)	$\tau_{Barostat}$(fs)	Dipoles?
Relax	1273	10 ps	0.25	100	500	X
Anneal1	1273	40 ps	1.0	100	500	✓
Anneal2	1373	40 ps	1.0	100	500	✓
Anneal3	1500	40 ps	1.0	100	500	✓
Anneal4	1640	40 ps	1.0	100	500	✓
Anneal5	1500	40 ps	1.0	100	500	✓
Anneal6	1373	40 ps	1.0	100	500	✓
Anneal7	1273	40 ps	1.0	100	500	✓
Equil	Target Temp.	1 ns	1.0	500	2500	✓
Prod	Target Temp.	2 ns	1.0	500	2500	✓

Frames were chosen from the PIM production run with the experimentally determined density. Specifically, for pure $LaCl_3$, a frame with density of 3.14 g/cm^3 as measured by Liu et. al.[11] was

used, and for $LaCl_3$-NaCl (50-50%) a value of 2.53 g/cm^3 from a fit of data in the same paper was used; these values match our newest experimental measurements in Figure 2 reasonably well.

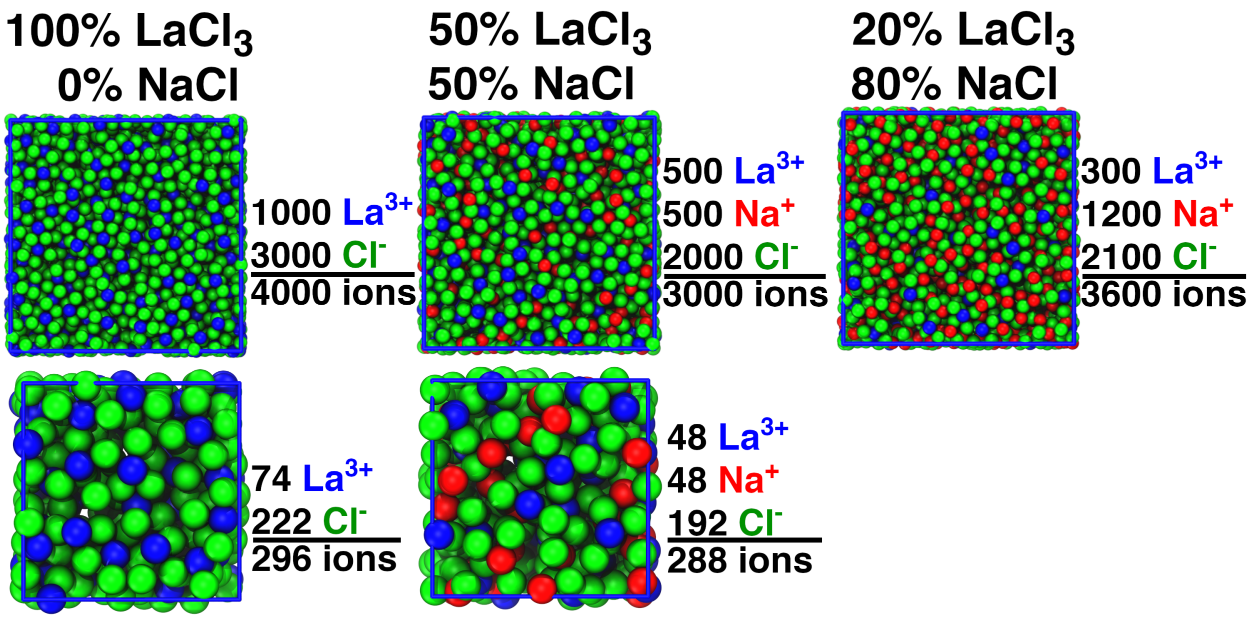

Figure S3: Characteristic snapshots at 900 °C and ion number information. Upper panel are equilibrated snapshots for PIM simulations whereas bottom panel are PIM-equilibrated snapshots used as initial configurations for first principles MD.

AIMD simulations were performed using the PBE exchange-correlation functional[13–16] and Grimme's D3 dispersion correction[17] implemented through the Quickstep[18] module of the CP2K 6.1 package.[19,20] The MOLOPT basis set[21] of double zeta valence plus polarization (DZVP-MOLOPT) in conjunction with Goedecker-Teter-Hutter (GTH) pseudopotentials[22] were applied for all ions, including new pseudopotentials and basis sets for La.[23] Additionally, we employed a valence triple-zeta basis set with two sets of polarization functions and a set of diffuse functions for light elements (TZV2P-MOLOPT[21]). A 800 Ry cutoff for the plane wave basis (CUTOFF), a 80 Ry cutoff of a reference grid (REL_CUTOFF), and a 1.0 fs timestep were used. The orbital transformation method was employed with a FULL_ALL preconditioner and a conjugate gradient minimizer to achieve and accelerate the SCF convergence. A Nosé-Hoover chain thermostat[24] with a velocity rescaling time constant of 1.0 picoseconds (ps) was used for the temperature coupling. Trajectories of at least 150 ps in length at 900 °C were generated using the DZVP basis set.

Then, we switched to the TZV2P basis set and restarted the AIMD simulations for another 100 ps. The last 60 ps from each run were used for calculating structural parameters, Raman spectra, and free energy profiles.

S1.5 Polarizable Ion Model (PIM) Simulations

The PIM has been used with various functional forms in the molten salt literature. Forcefields from the work of Madden, Wilson and Hutchinson, which for $LaCl_3$ originated in the early-to-mid 1990s, have enabled many important studies across various different ionic systems.[25,26]

The PIM force-field uses a sum of a Coulombic electrostatic potential, a Born-Mayer-Huggins (BMH) pair potential consisting of a repulsion and two dispersion terms, and an additional polarization term as shown below in Equation S3 and as coded in the software MetalWalls (release 20.05).[27]

$$\phi^{\text{total}} = \sum_{i}\sum_{j>i}\left(\frac{1}{4\pi\varepsilon_0}\frac{q_i q_j}{r_{ij}} + B_{ij} exp(-\alpha_{ij} r_{ij}) - f_6^{ij}(r_{ij})\frac{C_6^{ij}}{(r_{ij}^6)} - f_8^{ij}(r_{ij})\frac{C_8^{ij}}{(r_{ij}^8)}\right) + \phi^{\text{pol}}. \tag{S3}$$

q_i, is the charge of particle i, r_{ij} is the distance between particle i and particle j, B_{ij} and α_{ij} are repulsion parameters, C_6^{ij} and C_8^{ij} are dipole-dipole and dipole-quadrupole dispersion parameters respectively, and f_6^{ij} and f_8^{ij} are Tang-Toennies damping functions. The polarization potential, ϕ^{pol} consists of the usual charge-dipole, dipole-charge, dipole-dipole, and self term as shown in Equation S4.

$$\begin{aligned}\phi^{\text{pol}} = &\sum_{i}\sum_{j>i}\left(f_4^{ij}(r_{ij})\frac{q_i\left(\mathbf{r}_{ij}\cdot\boldsymbol{\mu}_j\right)}{r_{ij}^3} - f_4^{ji}(r_{ij})\frac{q_j\left(\boldsymbol{\mu}_i\cdot\mathbf{r}_{ij}\right)}{r_{ij}^3}\right)\\ &+\sum_{i}\sum_{j>i}\left(\frac{\boldsymbol{\mu}_i\cdot\boldsymbol{\mu}_j}{r_{ij}^3} - \frac{3(\mathbf{r}_{ij}\cdot\boldsymbol{\mu}_j)(\mathbf{r}_{ij}\cdot\boldsymbol{\mu}_i)}{r_{ij}^5}\right)\\ &+\sum_{i}\left(\frac{|\boldsymbol{\mu}_i|^2}{2\alpha_i}\right)\end{aligned} \tag{S4}$$

The Tang-Toennies damping functions[28] shown in Equation S5 are used to damp both the disper-

sion interactions in the BMH pair potential as well as the charge-dipole interactions.

$$f_n^{ij}(r_{ij}) = 1 - c_n^{ij} * exp(-b_n^{ij} r_{ij}) \sum_{k=0}^{n} \frac{(b_n^{ij} r_{ij})^k}{k!}. \tag{S5}$$

Similarly to works on pure $LaCl_3$ by Hutchinson et. al.,[26] $LaCl_3$-LiCl-KCl mixtures by Salanne et. al.,[29] and $MgCl_2$-KCl mixtures by Wu et. al,[2,30] who separately note that the polarizability of multivalent cations is not very important with regards to structure, this study also neglects multivalent cation polarization. Table S4 lists the charges and ionic polarizabilities of each ion used in this study.

Table S4: Ionic polarizabilies and charges

Ion	q(e)	α ($Bohr^3$)
Na^+	+1.000	0.9[31]
La^{3+}	+3.000	0[29]
Cl^-	-1.000	20.0[31]

For polarizable cation-anion interactions, there are up to three additional parameters controlling the charge-dipole interaction (see Table S5).

Table S5: Charge-Dipole Damping Parameters

Ion-Pair	$b_4^{ij}=b_4^{ji}$	c_4^{ij}	c_4^{ji}	Ref.
Na-Cl	1.760	3.000	0.697	[31]
La-Cl	1.258	1.000	N/A	[29]

As shown previously in the literature,[26,31] cation-cation BMH repulsion parameters are considered to have negligible effect when compared to the Coulombic repulsion in alkali halides; this is even more-so for multivalent cations. In addition, cross cation-cation dispersion parameters are often taken to be the same (see for example, La^{3+}-Na^+ and La^{3+}-La^{3+} parameters proposed by Glover et. al.[32] or the La^{3+}-La^{3+} and La^{3+}-K^+ proposed by Salanne[29]). With these considerations, a list of parameters adapted for our simulations are provided in Table S6, with the dispersion damping parameters c_6^{ij}=1 and c_8^{ij}=1 for all interactions.

Table S6: BMH Potential Parameters

Ion-Pair	α_{ij}	B_{ij}	C_6^{ij}	C_8^{ij}	b_6^{ij}	b_8^{ij}	Ref.
Na^+-Na^+	5.000	1.0	11.7	51.8	1.70	1.70	[31]
La^{3+}-Na^+	3.000	10.0	34.8	94.6	1.50	1.00	*
La^{3+}-La^{3+}	3.000	15.0	47.70	100.0	1.50	1.00	[29]
Na^+-Cl^-	1.726	67.5	47.4	187.3	1.70	1.70	[31]
La^{3+}-Cl^-	1.800	450.0	97.22	600.0	1.50	1.00	[29]
Cl^--Cl^-	1.797	275.1	140.0	280.0	1.70	1.70	[31]

* Taken to be the same as for La^{3+}-K^+ in reference 29.

Initial ionic configurations for each salt were generated using the "PACKMOL" package[33] and the equilibration protocol which was similar to that used in references 2 and 30 is provided in Table S3; target temperatures are provided in Table S1. All PIM runs were simulated in the isothermal-isobaric (NPT)[34] ensemble at 1.0 bar; temperature and pressure were controlled using the Nosé-Hoover[24,35] thermostat and barostat with chain lengths of 5, and relaxation times (τ) listed in Table S3. Simulations were carried out in 3D-periodically replicated boxes using the Ewald summation as coded in MetalWalls (release 20.05).[27,36,37] The 3D Ewald summation used a real-space summation tolerance of 1.63e-5 and reciprocal-space summation tolerance of 1.0e-7. Real-space cutoffs of 22.677 Bohr ($\approx$12.0 Å) and 12.0 Bohr ($\approx$6.35 Å) were used for boxes meant for classical and first principles calculations respectively. The induced dipoles were calculated at each timestep of the simulation by using a self-consistent conjugate gradient minimization with a convergence threshold of 1.0e-7. In the absence of a dedicated energy minimization tool in Metalwalls, a short non-polarizable run was used to briefly relax the random ionic configuration used as input (see Table S3). A 2 ns production run was used to calculate all averaged properties including radial distribution functions (RDFs), and corresponding X-ray S(q)s. A visual comparison (not to scale) between larger boxes meant for our classical studies and smaller PIM-equilibrated boxes used as input for AIMD is shown in Figure S3.

S1.6 Calculated Properties from Simulations

S1.6.1 Structure Functions

The total X-ray S(q) was calculated from simulation trajectories based on the following equation:

$$S(q) = \frac{\rho_0 \sum_i \sum_j \chi_i \chi_j f_i(q) f_j(q) \int_0^\infty 4\pi r^2 \left(g_{ij}(r) - 1\right) \frac{\sin(qr)}{qr} dr}{\left[\sum_v \chi_v f_v(q)\right]^2} \tag{S6}$$

where ρ_0 is the average atomic number density of the system, χ_i and χ_j are the mole fractions of ionic species i and j, $f_i(q)$ and $f_j(q)$ are the X-ray ionic form factors[9] for species i and j, and $g_{ij}(r)$ is the pair radial distribution function for species i and j.

The total X-Ray S(q) is decomposed into Faber-Ziman partial structure factor subcomponents:

$$S(q) = \sum_i \sum_{j \geq i} S_{ij}(q) \tag{S7}$$

where

$$S_{ii}(q) = \frac{\rho_0 \chi_i \chi_i f_i(q) f_i(q) \int_0^\infty 4\pi r^2 \left(g_{ii}(r) - 1\right) \frac{\sin(qr)}{qr} dr}{\left[\sum_v \chi_v f_v(q)\right]^2} \tag{S8}$$

and

$$S_{ij}(q) = 2 * \frac{\rho_0 \chi_i \chi_j f_i(q) f_j(q) \int_0^\infty 4\pi r^2 \left(g_{ij}(r) - 1\right) \frac{\sin(qr)}{qr} dr}{\left[\sum_v \chi_v f_v(q)\right]^2} \tag{S9}$$

S1.6.2 Coordination Numbers

If r_i is the distance between the ith Cl^- and a central La^{3+} and $r^\dagger$ represents the location of the boundary of the first chloride coordination shell obtained from the first minimum of the La^{3+}-Cl^- g(r), we define the Cl^- coordination number of a La^{3+} (CN_{La}) as:

$$CN_{La} = \sum_{i=1}^{N_{Cl}} \frac{1 - \left(\frac{r_i}{r^\dagger}\right)^{12}}{1 - \left(\frac{r_i}{r^\dagger}\right)^{24}} = \sum_{i=1}^{N_{Cl}} f_i^{Cl} \tag{S10}$$

were, f $(0 < f < 1)$ is a function allowing for smooth transitions of Cl^- across the boundary of the first chloride solvation shell; N_{Cl} is the total number of Cl^- ions. Likewise, the La^{3+} coordination number of a Cl^- ion (CN_{Cl}, i.e., number of La^{3+} around a Cl^- ion) is defined as:

$$CN_{Cl} = \sum_{i=1}^{N_{La}} \frac{1 - \left(\frac{r_i}{r^\dagger}\right)^{12}}{1 - \left(\frac{r_i}{r^\dagger}\right)^{24}} = \sum_{i=1}^{N_{La}} f_i^{La} \tag{S11}$$

where N_{La} is the total number of La^{3+} ions. CN_{La} and CN_{Cl} vary along a simulation due to thermal fluctuations and different metastable CN states are visited. We will see in the results section that CN_{La}=6,7,8,9 and CN_{Cl}=0,1,2,3,4. The population for each of these states are computed by defining them respectively through the following ranges:

(i) $5.5 < CN_{La} < 6.5$, $6.5 < CN_{La} < 7.5$, $7.5 < CN_{La} < 8.5$, and $8.5 < CN_{La} < 9.5$

(ii) $0.0 < CN_{Cl} < 0.5$, $0.5 < CN_{Cl} < 1.5$, $1.5 < CN_{Cl} < 2.5$, $2.5 < CN_{Cl} < 3.5$, and $3.5 < CN_{Cl} < 4.5$

It should be noted that, while it is common practice to integrate g(r) in order to determine an average coordination number, Equations S10 and S11 help us better understand heterogeneity and metastability in coordination. Associated free energy profiles can be straightforwardly determined from the CN distributions ($P(CN)$) though the relation: $W(CN) = -k_B T ln(P(CN))$, where T is the system temperature and k_B is the Boltzmann constant.

S1.6.3 2D Free Energy Calculations

f_i^{Cl} in Equation S11 is the contribution of the ith Cl^- to the coordination structure of a La^{3+} ion. Therefore, we can define functions f_{ij}^{Cl} and f_{ik}^{Cl} which are the contributions of the ith Cl^- to the coordination structures of the jth and k^{th} La^{3+} ions respectively. The number of Cl^- ions shared between these two La^{3+} ions is given by:

$$n_{La}^{Cl} = \sum_{i=1}^{N_{Cl}} f_{ij} f_{ik} \tag{S12}$$

Likewise, the number of La^{3+} ions coordinating with two different La^{3+} ions (j and k) through chloride sharing (n_{La}^{La}) is given by:

$$n_{La}^{La} = \sum_{i=1(\neq j,\neq k)}^{N_{La}} f_{ij} f_{ik} \tag{S13}$$

Herein, f is obtained by using $r^{\dagger}$ from the first minimum of the La^{3+}-La^{3+} g(r). The 2D free energy surfaces, $W\left(r_{\text{La-La}}, n_{\text{La}}^{\text{Cl}}\right)$ and $W\left(r_{\text{La-La}}, n_{\text{La}}^{\text{La}}\right)$, which can be used to resolve different metastable states describing local –La—La— interactions and configurations (such as edge-sharing, corner-sharing, and cross-network), are computed from the joint probability distribution (P) of r and n: $W\left(r_{\text{La-La}}, n_{\text{La}}^{\text{Cl}}\right) = -kBTln(P(r_{\text{La-La}}, n_{\text{La}}^{\text{Cl}}))$ and $W\left(r_{\text{La-La}}, n_{\text{La}}^{\text{La}}\right) = -kBTln(P(r_{\text{La-La}}, n_{\text{La}}^{\text{La}}))$.

S1.6.4 Raman Spectroscopy

For Raman calculations, we followed the same approach as discussed in our previous study.[3] Briefly, the Raman spectra were computed by Fourier transformation of time autocorrelation functions involving the appropriate polarizability tensor components of the full periodic simulation cell. The polarizability tensor was obtained from the induced dipole moments resulting from an applied periodic electric field (E = 5.0E-4 a.u.) in all three directions. Dipole moments were computed directly from the wave function, using the correct quantum chemical operator from modern theory of polarization (Berry phase approach), as implemented in CP2K. This approach does not make any assumptions about the polarizability model and can naturally account for any charge transfer and many other cooperative effects which cannot be captured well in terms of the polarizability of the individual ions. The polarizability tensor sampled every 4 fs was used as an input to the TRAVIS software[38] to generate the time autocorrelation functions and compute the raw Raman spectra in the polarized and depolarized scattering geometry ($I_{\sigma}(\omega)$). A correlation depth of 0.8 ps was found to give the optimal signal to noise ratio. A reduced Raman spectrum $R_{\sigma}(\omega)$[39] is

obtained from $I_\sigma(\omega)$ as

$$R_\sigma(\omega) = (\omega_L \pm \omega)^{-4}\,\omega\,(n\,(\omega,T)+1)^{-1}\,I_\sigma(\omega) \tag{S14}$$

Here, ω_L is the frequency of the incident laser light and $n(\omega,T)$ is the Boltzmann population factor for Stokes Raman scattering, $n(\omega,T) = exp(\hbar\omega/k_B T - 1) - 1$, where $\hbar$ and k_B are the reduced Planck and Boltzmann constants. The reduced representation of the Raman spectrum removes the temperature effect and the fourth power correction for the wavelength dependence of the scattering intensity, making $R_\sigma(\omega)$ dependent only on the components of the polarizability tensor.

S2 Supplemental Figures and Tables

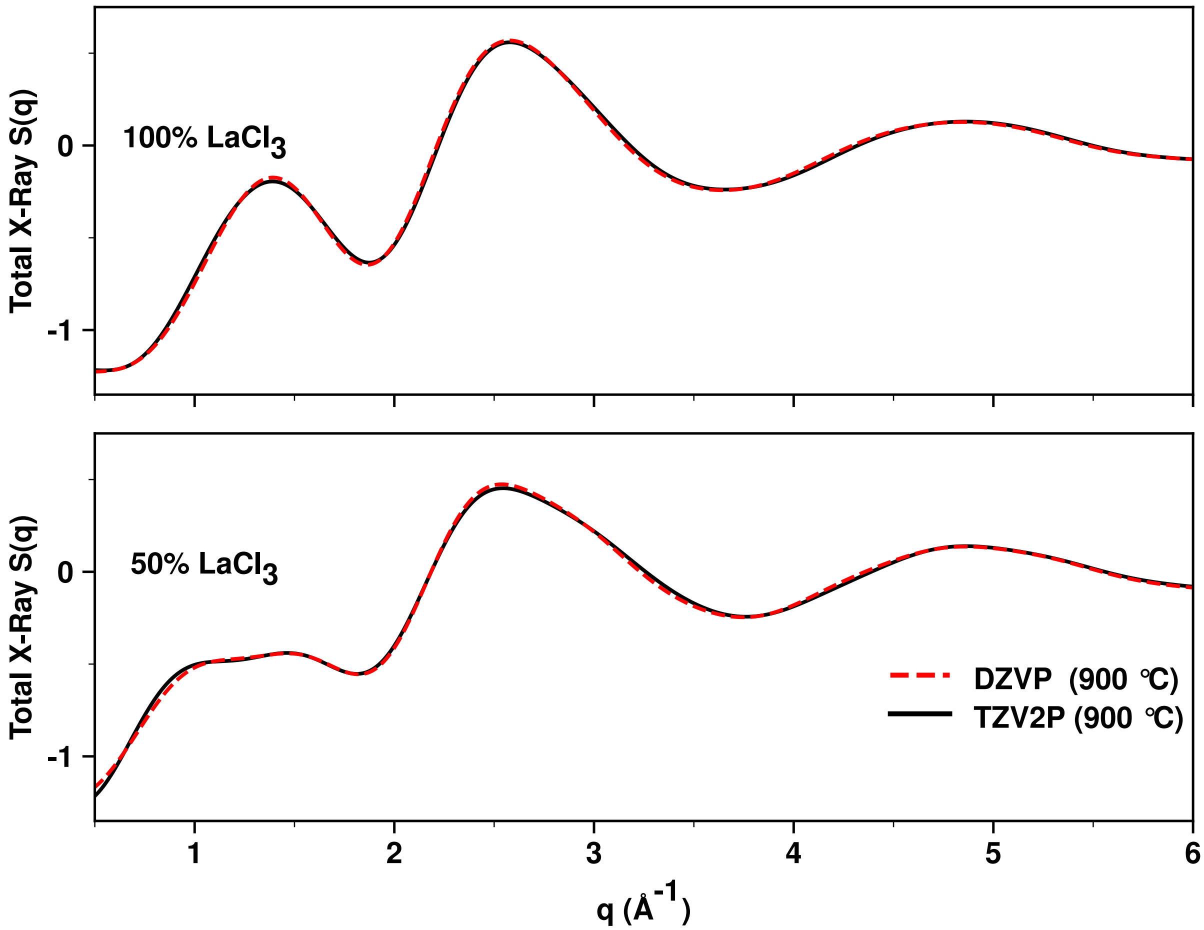

Figure S4: Comparison of X-Ray S(q) for $LaCl_3$ and the 50 % $LaCl_3$-NaCl mixture using the TZV2P and DZVP basis sets showing that for practical purposes the two give the same results.

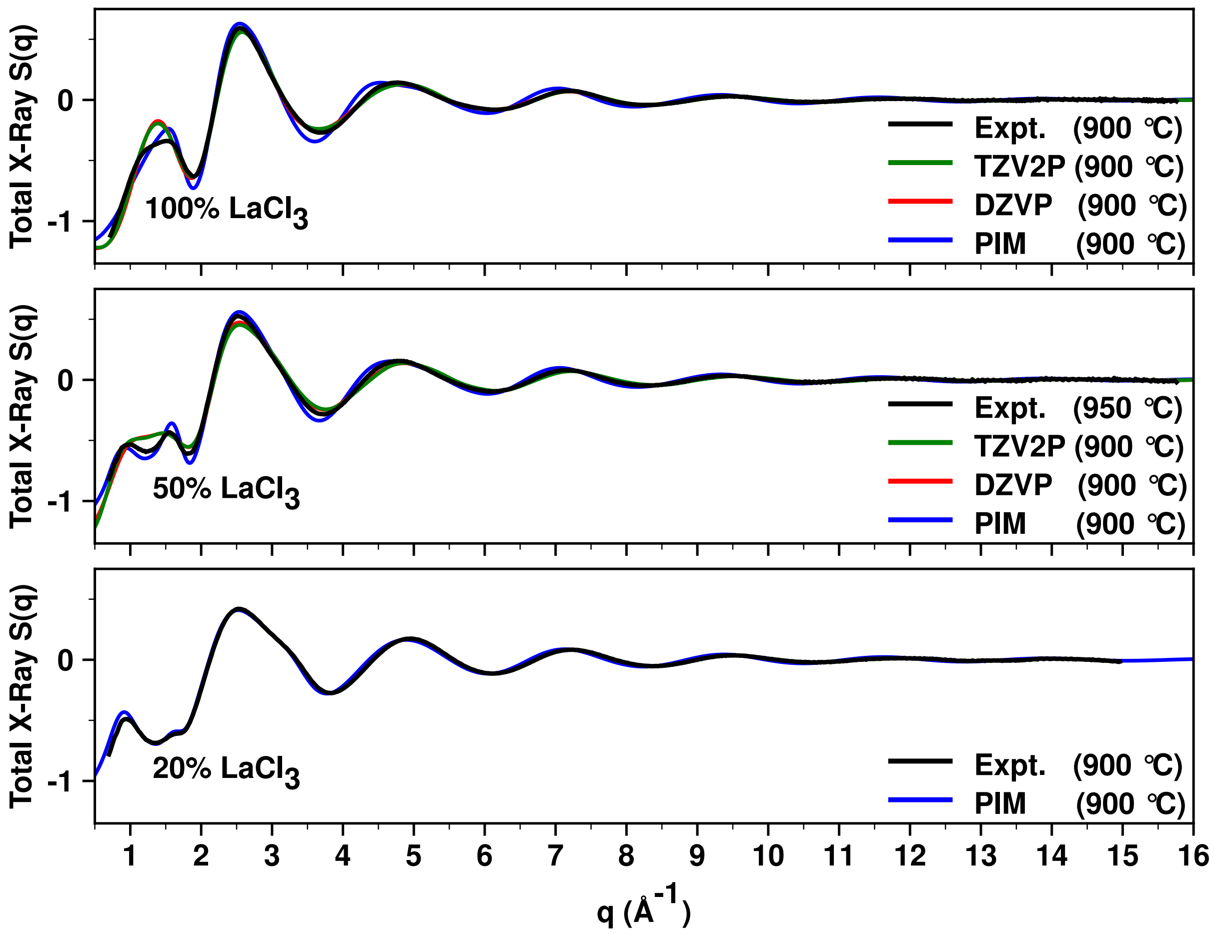

Figure S5: Comparison of simulated and full-range experimental X-Ray S(q) for $LaCl_3$ and $LaCl_3$-NaCl mixtures.

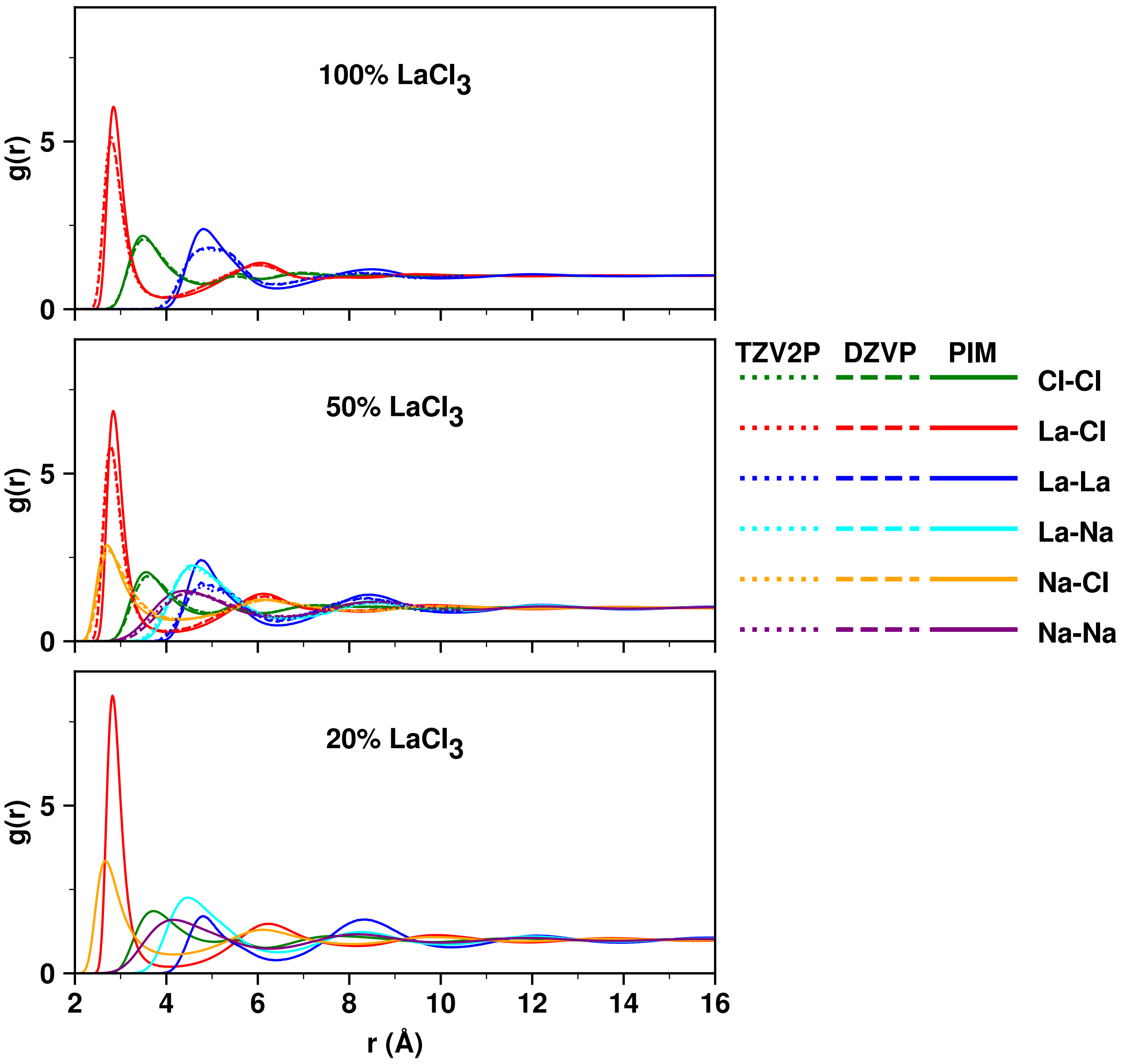

Figure S6: PIM and AIMD Simulated RDFs for pure $LaCl_3$ and its mixtures with NaCl at 900 °C for systems with number of ions described in Figure S3.

Table S7: AIMD and PIM populations (%) of different La-Cl coordination states, (left: CN_{La}) and (right: CN_{Cl}) at 900 °C.

Salt	Simulation Type	CN_{La}				CN_{Cl}				
		6	7	8	9	0	1	2	3	4
100% $LaCl_3$	PIM	0.32	12.94	57.40	29.34	0.00	0.12	37.48	56.94	5.46
	DZVP	1.63	31.36	57.69	9.32	0.00	0.84	47.66	49.14	2.36
	TZV2P	1.66	33.26	56.63	8.45	0.00	0.83	48.63	48.36	2.18
50% $LaCl_3$	PIM	2.00	30.24	57.03	10.73	0.74	26.27	55.36	16.95	0.68
	DZVP	14.26	58.81	25.97	0.96	0.45	34.84	54.45	10.1	0.16
	TZV2P	14.48	60.29	24.52	0.71	0.67	35.11	54.17	9.86	0.19
20% $LaCl_3$	PIM	13.74	56.86	28.38	1.02	19.51	61.04	18.49	0.96	0.00

References

(1) Toby, B. H.; Von Dreele, R. B. GSAS-II: the genesis of a modern open-source all purpose crystallography software package. *Journal of Applied Crystallography* **2013**, *46*, 544–549.

(2) Wu, F. et al. Elucidating ionic correlations beyond simple charge alternation in molten $MgCl_2$–KCl mixtures. *J. Phys. Chem. Lett.* **2019**, *10*, 7603–7610.

(3) Roy, S.; Brehm, M.; Sharma, S.; Wu, F.; Maltsev, D. S.; Halstenberg, P.; Gallington, L. C.; Mahurin, S. M.; Dai, S.; Ivanov, A. S.; Margulis, C. J.; Bryantsev, V. S. Unraveling Local Structure of Molten Salts via X-ray Scattering, Raman Spectroscopy, and Ab Initio Molecular Dynamics. *The Journal of Physical Chemistry B* **2021**, *125*, 5971–5982.

(4) Roy, S.; Sharma, S.; Karunaratne, W. V.; Wu, F.; Gakhar, R.; Maltsev, D. S.; Halstenberg, P.; Abeykoon, M.; Gill, S. K.; Zhang, Y.; Mahurin, S. M.; Dai, S.; Bryantsev, V. S.; Margulis, C. J.; Ivanov, A. S. X-ray scattering reveals ion clustering of dilute chromium species in molten chloride medium. *Chemical Science* **2021**, *12*, 8026–8035.

(5) Chupas, P. J.; Chapman, K. W.; Kurtz, C.; Hanson, J. C.; Lee, P. L.; Grey, C. P. A versatile sample-environment cell for non-ambient X-ray scattering experiments. *Journal of Applied Crystallography* **2008**, *41*, 822–824.

(6) Qiu, X.; Thompson, J. W.; Billinge, S. J. L. PDFgetX2: a GUI-driven program to obtain the pair distribution function from X-ray powder diffraction data. *Journal of Applied Crystallography* **2004**, *37*, 678–678.

(7) Billinge, T. E.; S., *Underneath the Bragg peaks: Structural Analysis of Complex Materials*; Elsevier, 2012; Vol. 16.

(8) Fischer, H. E.; Barnes, A. C.; Salmon, P. S. Neutron and x-ray diffraction studies of liquids and glasses. *Reports on Progress in Physics* **2006**, *69*, 233–299.

(9) Brown, P. J.; Fox, A. G.; Maslen, E. N.; O'Keefe, M. A.; Willis, B. T. M. Intensity of diffracted intensities. In *International Tables for Crystallography*, Vol. C, Chapter 6.1, International Union of Crystallography, 2006; pp. 554-595.

(10) Zissi, G. D.; Chrissanthopoulos, A.; Papatheodorou, G. N. Vibrational modes and structure of the LaCl3-CsCl melts. *Vibrational Spectroscopy* **2005**, *40*, 110–117.

(11) Liu, G.; Toguri, J. M.; Stubina, N. M. Surface tension and density of the molten LaCl3–NaCl binary system. *Canadian Journal of Chemistry* **1987**, *65*, 2779–2782.

(12) Duemmler, K.; Lin, Y.; Woods, M.; Karlsson, T.; Gakhar, R.; Beeler, B. Evaluation of thermophysical properties of the LiCl-KCl system via ab initio and experimental methods. *Journal of Nuclear Materials* **2022**, *559*.

(13) Perdew, J. P.; Burke, K.; Ernzerhof, M. Generalized Gradient Approximation Made Simple. *Physical Review Letters* **1996**, *77*, 3865–3868.

(14) Perdew, J. P.; Burke, K.; Ernzerhof, M. Perdew, Burke, and Ernzerhof Reply. *Physical Review Letters* **1998**, *80*, 891–891.

(15) Zhang, Y.; Yang, W. Comment on "Generalized Gradient Approximation Made Simple". *Physical Review Letters* **1998**, *80*, 890–890.

(16) Perdew, J. P.; Ruzsinszky, A.; Csonka, G. I.; Vydrov, O. A.; Scuseria, G. E.; Constantin, L. A.; Zhou, X.; Burke, K. Restoring the Density-Gradient Expansion for Exchange in Solids and Surfaces. *Physical Review Letters* **2008**, *100*.

(17) Grimme, S.; Antony, J.; Ehrlich, S.; Krieg, H. A consistent and accurate <i>ab initio</i> parametrization of density functional dispersion correction (DFT-D) for the 94 elements H-Pu. *The Journal of Chemical Physics* **2010**, *132*, 154104.

(18) Vandevondele, J.; Krack, M.; Mohamed, F.; Parrinello, M.; Chassaing, T.; Hutter, J. Quickstep: Fast and accurate density functional calculations using a mixed Gaussian and plane waves approach. *Computer Physics Communications* **2005**, *167*, 103–128.

(19) Hutter, J.; Iannuzzi, M.; Schiffmann, F.; VandeVondele, J. CP2K: atomistic simulations of condensed matter systems. *WIREs Comput. Mol. Sci.* **2014**, *4*, 15–25.

(20) Kühne, T. D. et al. CP2K: An electronic structure and molecular dynamics software package - Quickstep: Efficient and accurate electronic structure calculations. *The Journal of Chemical Physics* **2020**, *152*, 194103.

(21) Vandevondele, J.; Hutter, J. Gaussian basis sets for accurate calculations on molecular systems in gas and condensed phases. *The Journal of Chemical Physics* **2007**, *127*, 114105.

(22) Goedecker, S.; Teter, M.; Hutter, J. Separable dual-space Gaussian pseudopotentials. *Physical Review B* **1996**, *54*, 1703–1710.

(23) Lu, J.-B.; Cantu, D. C.; Nguyen, M.-T.; Li, J.; Glezakou, V.-A.; Rousseau, R. Norm-Conserving Pseudopotentials and Basis Sets To Explore Lanthanide Chemistry in Complex Environments. *Journal of Chemical Theory and Computation* **2019**, *15*, 5987–5997.

(24) Nosé, S. A unified formulation of the constant temperature molecular dynamics methods. *The Journal of Chemical Physics* **1984**, *81*, 511–519.

(25) Hutchinson, F.; Rowley, A. J.; Walters, M. K.; Wilson, M.; Madden, P. A.; Wasse, J. C.; Salmon, P. S. Structure of Molten MCl_3 Systems from a Polarizable Ion Simulation Model. *The Journal of Chemical Physics* **1999**, *111*, 2028–2037.

(26) Hutchinson, F.; Wilson, M.; Madden, P. A. A Unified Description of MCI3 systems with a Polarizable Ion Simulation Model. *Molecular Physics* **2001**, *99*, 811–824.

(27) Marin-Laflèche, A.; Haefele, M.; Scalfi, L.; Coretti, A.; Dufils, T.; Jeanmairet, G.; Reed, S.; Serva, A.; Berthin, R.; Bacon, C.; Bonella, S.; Rotenberg, B.; Madden, P.; Salanne, M. MetalWalls: A classical molecular dynamics software dedicated to the simulation of electrochemical systems. *Journal of Open Source Software* **2020**, *5*.

(28) Tang, K. T.; Toennies, J. P. An improved simple model for the van der Waals potential based on universal damping functions for the dispersion coefficients. *The Journal of Chemical Physics* **1984**, *80*, 3726–3741.

(29) Salanne, M.; Simon, C.; Turq, P.; Madden, P. A. Calculation of Activities of Ions in Molten Salts with Potential Application to the Pyroprocessing of Nuclear Waste. *The Journal of Physical Chemistry B* **2008**, *112*, 1177–1183.

(30) Wu, F.; Sharma, S.; Roy, S.; Halstenberg, P.; Gallington, L. C.; Mahurin, S. M.; Dai, S.; Bryantsev, V. S.; Ivanov, A. S.; Margulis, C. J. Temperature dependence of short and intermediate range order in molten $MgCl_2$ and its mixture with KCl. *J. Phys. Chem. B* **2020**, *124*, 2892–2899.

(31) Ishii, Y.; Kasai, S.; Salanne, M.; Ohtori, N. Transport coefficients and the Stokes–Einstein relation in molten alkali halides with polarisable ion model. *Molecular Physics* **2015**, *113*, 2442–2450.

(32) Glover, W. J.; Madden, P. A. Raman spectra of ionic liquids: A simulation study of LaCl3 and its mixtures with alkali chlorides. *The Journal of Chemical Physics* **2004**, *121*, 7293–7303.

(33) Martínez, L.; Andrade, R.; Birgin, E. G.; Martínez, J. M. PACKMOL: A package for building initial configurations for molecular dynamics simulations. *J. Comput. Chem.* **2009**, *30*, 2157–2164.

(34) Martyna, G. J.; Tobias, D. J.; Klein, M. L. Constant pressure molecular dynamics algorithms. *The Journal of Chemical Physics* **1994**, *101*, 4177–4189.

(35) Hoover, W. G. Canonical dynamics: Equilibrium phase-space distributions. *Phys Rev A Gen Phys* **1985**, *31*, 1695–1697.

(36) Ewald, P. P. Die Berechnung optischer und elektrostatischer Gitterpotentiale. *Annalen der Physik* **1921**, *369*, 253–287.

(37) Aguado, A.; Madden, P. A. Ewald summation of electrostatic multipole interactions up to the quadrupolar level. *The Journal of Chemical Physics* **2003**, *119*, 7471–7483.

(38) Brehm, M.; Thomas, M.; Gehrke, S.; Kirchner, B. TRAVIS—A free analyzer for trajectories from molecular simulation. *The Journal of Chemical Physics* **2020**, *152*, 164105.

(39) Papatheodorou, G. N.; Yannopoulos, S. N. *Light Scattering from Molten Salts: Structure and Dynamics*; Springer Netherlands, 2002; pp 47–106.
